# Revealing prognostic insights of programmed cell death (PCD)-associated genes in advanced non-small cell lung cancer

**DOI:** 10.18632/aging.205807

**Published:** 2024-05-08

**Authors:** Weiwei Dong, He Zhang, Li Han, Huixia Zhao, Yue Zhang, Siyao Liu, Jiali Zhang, Beifang Niu, Wenhua Xiao

**Affiliations:** 1 Senior Department of Oncology, The Fifth Medical Center of PLA General Hospital, Beijing 100071, P.R. China; 2 Department of Oncology, The Forth Medical Center of PLA General Hospital, Beijing 100048, P.R. China; 3 Beijing ChosenMed Clinical Laboratory Co. Ltd., Beijing 100176, P.R. China; 4 Computer Network Information Center, Chinese Academy of Sciences, Beijing 100083, P.R. China; 5 University of the Chinese Academy of Sciences, Beijing 100049, P.R. China

**Keywords:** advanced NSCLC, programmed cell death-related genes, PRAN risk model, RT-qPCR, molecular docking

## Abstract

The management of patients with advanced non-small cell lung cancer (NSCLC) presents significant challenges due to cancer cells’ intricate and heterogeneous nature. Programmed cell death (PCD) pathways are crucial in diverse biological processes. Nevertheless, the prognostic significance of cell death in NSCLC remains incompletely understood. Our study aims to investigate the prognostic importance of PCD genes and their ability to precisely stratify and evaluate the survival outcomes of patients with advanced NSCLC. We employed Weighted Gene Co-expression Network Analysis (WGCNA), Least Absolute Shrinkage and Selection Operator (LASSO), univariate and multivariate Cox regression analyses for prognostic gene screening. Ultimately, we identified seven PCD-related genes to establish the PCD-related risk score for the advanced NSCLC model (PRAN), effectively stratifying overall survival (OS) in patients with advanced NSCLC. Multivariate Cox regression analysis revealed that the PRAN was the independent prognostic factor than clinical baseline factors. It was positively related to specific metabolic pathways, including hexosamine biosynthesis pathways, which play crucial roles in reprogramming cancer cell metabolism. Furthermore, drug prediction for different PRAN risk groups identified several sensitive drugs explicitly targeting the cell death pathway. Molecular docking analysis suggested the potential therapeutic efficacy of navitoclax in NSCLC, as it demonstrated strong binding with the amino acid residues of C-C motif chemokine ligand 14 (CCL14), carboxypeptidase A3 (CPA3), and C-X3-C motif chemokine receptor 1 (CX3CR1) proteins. The PRAN provides a robust personalized treatment and survival assessment tool in advanced NSCLC patients. Furthermore, identifying sensitive drugs for distinct PRAN risk groups holds promise for advancing targeted therapies in NSCLC.

## INTRODUCTION

According to the 8^th^ edition TNM staging proposed by the International Association for the Study of Lung Cancer (IASLC), lung cancer patients with stage IIIA or more advanced stages have been reported to exhibit a 5-year survival rate of less than 36% [1]. Recent global cancer data released in 2020 revealed that lung cancer remains the leading cause of cancer-related mortality [2]. This dismal outcome in lung cancers is due, in part to the fact that more than half of the patients, about 55%, presented with metastatic lung cancer at the time of diagnosis [3]. Among lung cancer cases, non-small cell lung cancer (NSCLC) accounts for approximately 85% of cases, with lung adenocarcinoma (LUAD) and lung squamous cell carcinoma (LUSC) being the most prevalent subtypes [1]. Survival outcomes are notably compromised for patients with advanced NSCLC, including those with metastatic or recurrent disease after initial definitive treatment, particularly in stages III and IV [4]. The significantly low survival rate in advanced NSCLC underscores the formidable challenges its intricate nature and heterogeneity pose.

The advent of immune checkpoint inhibitors (ICIs) has significantly transformed the treatment landscape of lung cancer, particularly for advanced metastatic NSCLC. PD-(L)1 expression, tumor mutational burden (TMB) level, and microsatellite instability/defective mismatch repair (MSI/dMMR) status have emerged as important indicators to screen patients for potential long-term benefits from ICI therapy (https://www.nccn.org/). However, the tumor immune microenvironment (TIME) presents complex and heterogeneous characteristics across tumor types, especially for advanced cancers [5]. Due to the effectiveness of ICI therapy in improving the survival benefits of advanced NSCLC patients, there is a continuous need to explore more precise molecular biomarkers that can indicate the efficacy of immunotherapy.

Recently, numerous prognosis signatures have been developed to stratify cancer patients accurately. In breast cancer, Oncotype DX and MammaPrint are two widely used prognostic and predictive biomarkers that assist in optimizing therapy decisions and evaluating prognosis. The MINDACT study demonstrated that patients classified as low risk of recurrence based on MammaPrint but high risk according to clinicopathological criteria achieved a 94.7% 5-year distant metastasis-free survival [6]. Wu et al. developed an immune-related prognosis signature comprising 21 immune-related genes, enabling more accurate survival risk stratification for early-stage LUAD [7]. Feng et al. developed a CD8+ T cell-related risk model to predict immunotherapy outcomes and evaluate the survival of stage III LUAD patients [8]. In conclusion, mRNA expression is closely associated with cancer patients’ prognosis and therapeutic efficacy.

Resisting cell death is a hallmark characteristic of cancer [9]. Programmed cell death (PCD) encompasses various pathways, including apoptosis, ferroptosis, and autophagy, and so on. These PCD pathways play pivotal roles in eliminating malignant and infected cells [10]. Epigenetic modifications can regulate these pathways, thus influencing tumor progression and treatment resistance [11]. Ferroptosis is a distinctive PCD pathway that regulates cell death by accumulating iron and lipid reactive oxygen species (ROS) within cells. It holds significant potential as a target for drug therapy. Ferroptosis inhibitors could regulate and activate P53 and its downstream genes and further induce ROS accumulation and ferroptosis [12, 13]. Ku et al. discovered that JI017 can induce cell autophagy and apoptosis by enhancing ROS levels, reducing tumor size in lung cancer. This remarkable application of cell death regulation in cancer therapy exemplifies the potential of this approach in treating malignant tumors [14]. Furthermore, the induction of autophagy has been demonstrated to effectively overcome the resistance to third-generation EGFR-TKI treatment in patients with NSCLC [15].

However, the implication of PCD pathway genes in the advanced NSCLC is still unknown. We need to elucidate the molecular significance of PCD-related genes in the tumor microenvironment and their correlation with patient survival outcomes and treatment efficacy assessment. This knowledge will enable better guidance for clinicians in precise evaluation. Furthermore, it can provide a theoretical basis for developing corresponding targeted drugs. This study also explored the prognosis value of PCD-related genes for survival and prediction ability for immunotherapy response in NSCLC. The Cancer Genome Atlas (TCGA) [16] dataset and Gene Expression Omnibus (GEO, https://www.ncbi.nlm.nih.gov/geo/) [17] database were utilized to develop and validate the performance of the model. As a result, seven genes associated with prognosis were identified. A risk model was constructed to assess the prognosis and accurately stratify advanced NSCLC patients to enhance survival outcomes.

## RESULTS

### Multi-omics analysis of PCD-related genes

**Figure 1:**
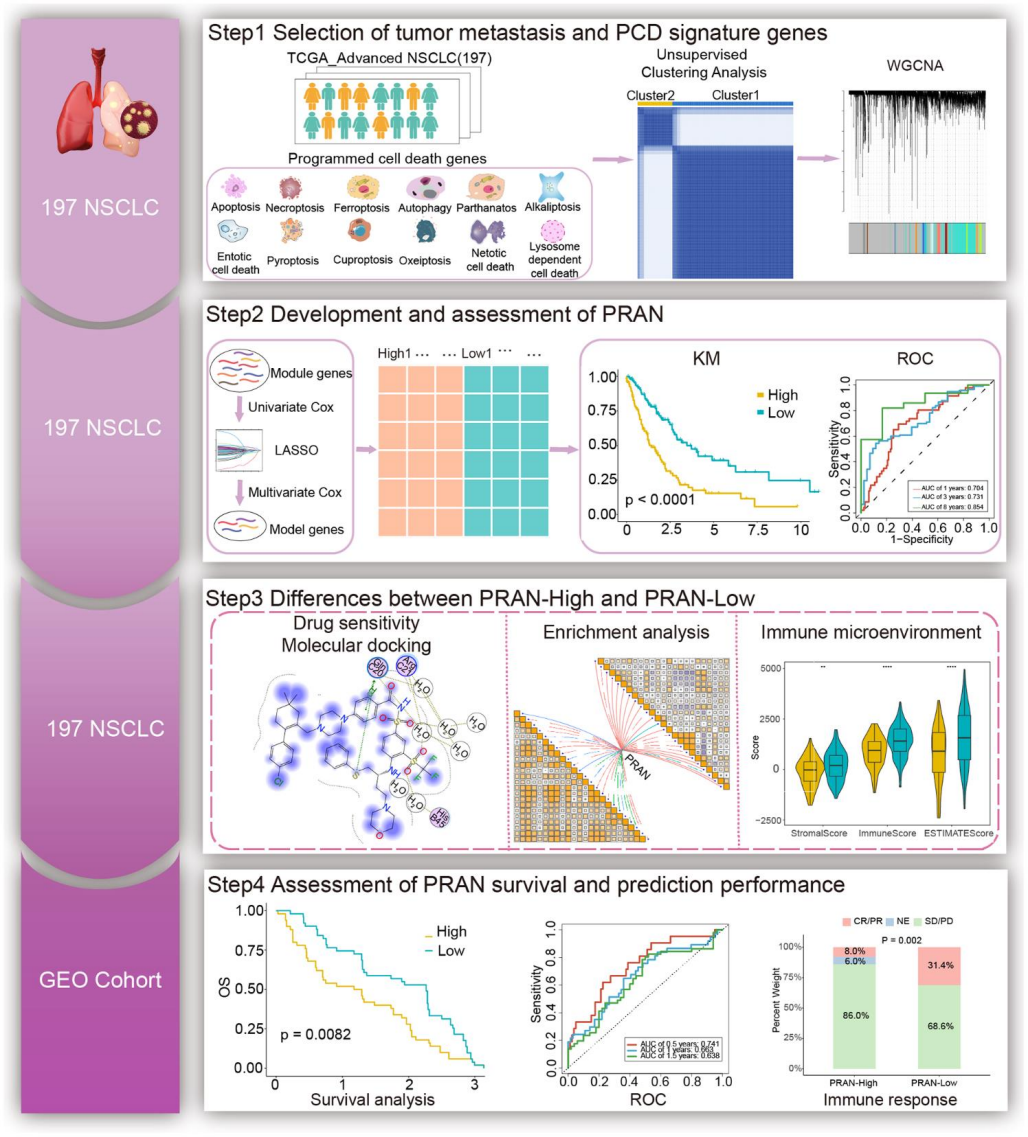
The workflow of the study.

**Figure 2:**
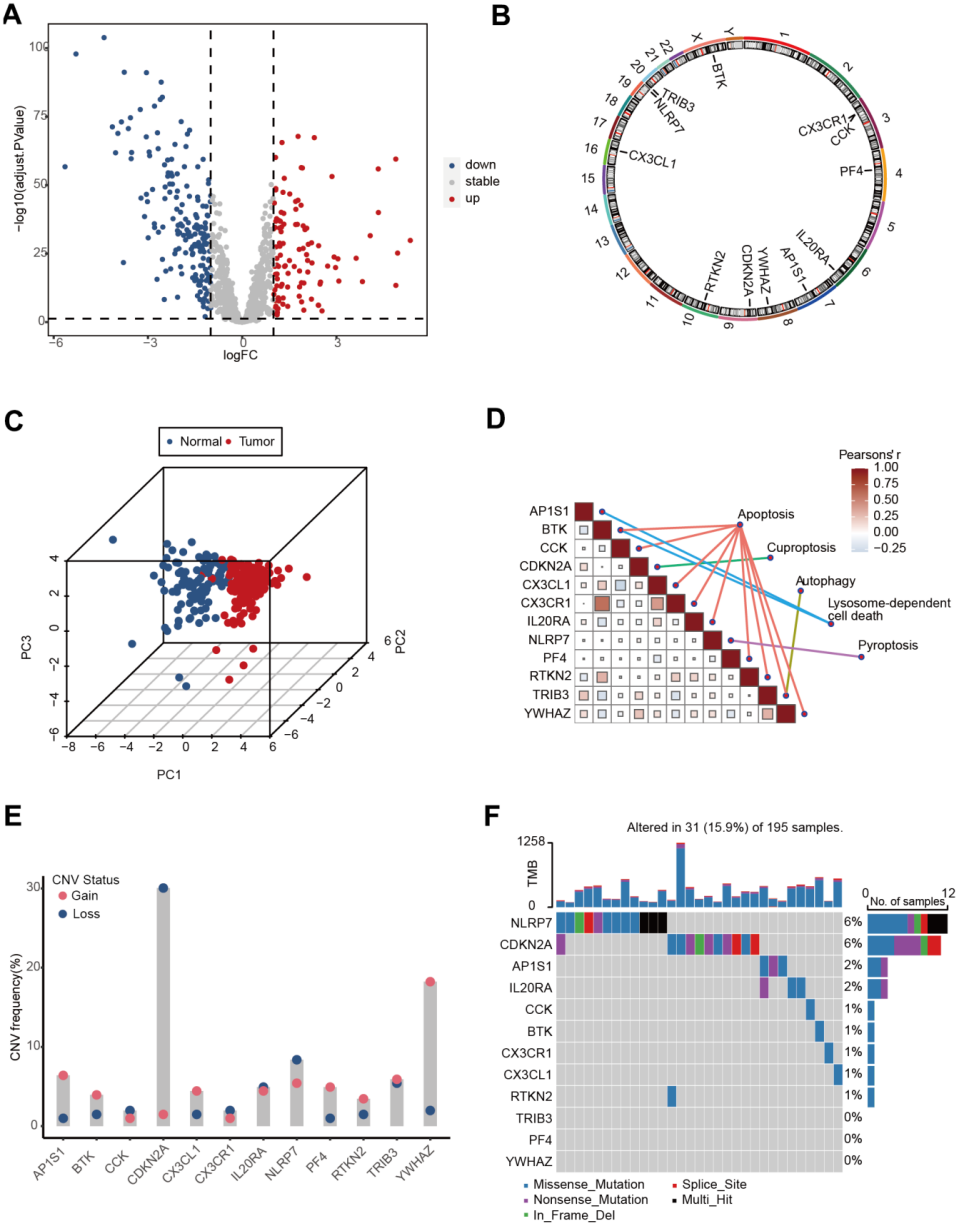
**The landscape of genetic and transcriptional alterations of PCD genes in TCGA-Advanced NSCLC.** (**A**) Volcano plot depicting the differential expression of PCD genes between tumor and normal samples. (**B**) Circos plot illustrating the chromosomal distribution of 12 prognosis related differential PCD genes. Each outer circle represents a chromosome, and the connecting lines display the genomic location of the PCD genes. (**C**) Principal Component Analysis (PCA) plot of 12 PCD-related genes. (**D**) Correlation analysis heatmap of 12 PCD-related genes in the TCGA-Advanced NSCLC dataset. The color scale represents the correlation coefficients, with red indicating positive correlation and blue indicating negative correlation. (**E**) Copy Number Variation (CNV) frequencies of 12 PCD-related genes. (**F**) Mutation frequencies of 12 PCD-associated differential genes in the TCGA-Advanced NSCLC cohort. The column height represents the frequency of mutations, and different types of mutations are distinguished by color.

The workflow for this study is shown in Figure 1. The PCD-related genes contained the essential regulatory genes of twelve PCD pathways collected from the studies of Zou et al. [18]. Finally, 1178 PCD-related genes were brought into the analysis (Supplementary Table 1). By comparing the expression profiles of 1178 genes associated with PCD between advanced NSCLC and normal tissues from the TCGA-Advanced dataset, we have successfully identified 278 differentially expressed genes (DEGs) (P adjust. < 0.05 and | log2FC | > 1). In the TCGA-Advanced NSCLC cohort, 105 genes were upregulated, and 173 genes were downregulated compared to normal tissues (Figure 2A).

The altered expression patterns of PCD-related genes in advanced NSCLC provide valuable insights. To assess the prognostic significance of these DEGs, we analyzed their correlation with the survival status of advanced NSCLC patients. Survival analysis revealed that 12 genes among all DEGs significantly influenced patient prognosis, including adaptor related protein complex 1 subunit sigma 1 (*AP1S1*), bruton tyrosine kinase (*BTK*), cholecystokinin (*CCK*), cyclin dependent kinase inhibitor 2A *(CDKN2A*), C-X3-C motif chemokine ligand 1 (*CX3CL1*), *CX3CR1*, interleukin 20 receptor subunit alpha (*IL20RA*), NLR family pyrin domain containing 7 (*NLRP7*), platelet factor 4 (*PF4*), rhotekin 2 (*RTKN2*), tribbles pseudokinase 3 (*TRIB3*), and tyrosine 3-monooxygenase/tryptophan 5-monooxygenase activation protein zeta (*YWHAZ*) (Supplementary Figure 1). The chromosomal positions of these 12 PCD genes are depicted in Figure 2B. Principal component analysis (PCA) revealed that the expression of the 12 PCD genes could effectively discriminate advanced NSCLC samples from normal samples (Figure 2C). These 12 PCD genes participated in various PCD processes, including apoptosis, cuproptosis, autophagy, pyroptosis, and lysosome-dependent cell death, and exhibited close interactions (Figure 2D).

Furthermore, copy-number alterations (CNA) analysis revealed frequent copy number variations changes in these 12 PCD genes, with a predominant occurrence of *YWHAZ* amplification and *CDKN2A* deletion (Figure 2E). Further exploration of somatic mutation of 12 PCD genes exhibited a 21% mutation rate in advanced NSCLC. Notably, *NLRP7* and *CDKN2A* showed the highest mutation frequency, accounting for 6% of the observed mutations (Figure 2F).

These findings indicated the potential utility of 12 PCD genes as prognostic markers in advanced NSCLC.

### Identification and enrichment analysis of PCD-related subtypes

**Figure 3:**
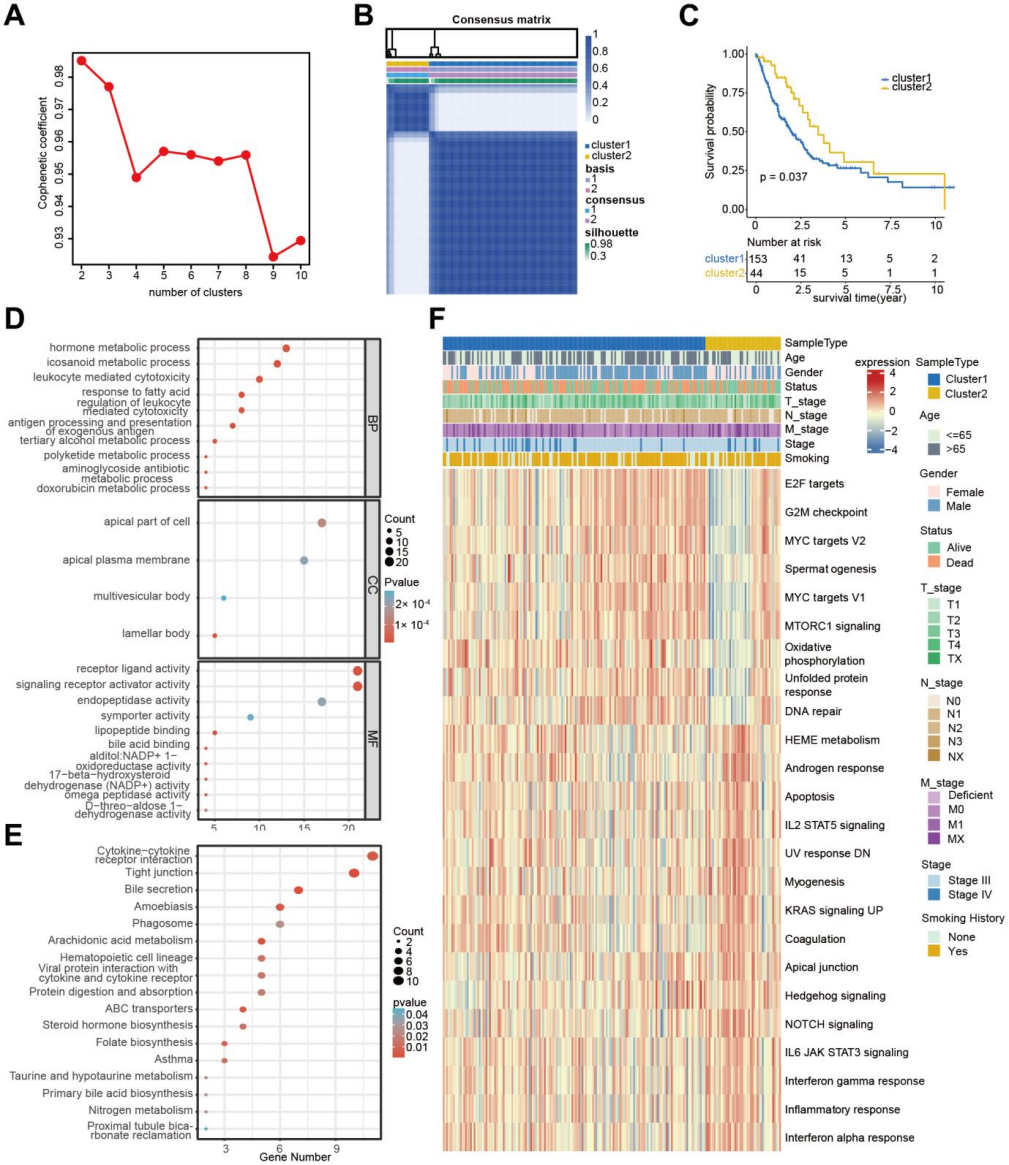
**Cluster analysis of 12 PCD-related genes in TCGA-Advanced NSCLC dataset.** (**A**) Non-negative Matrix Factorization (NMF) clustering of twelve PCD-related genes. The correlation coefficients at k = 2-10 are presented in the Figure. (**B**) Consistency plot illustrating the stability of NMF clustering results. (**C**) Kaplan-Meier (KM) survival curves of patients in PCD-related clusters. (**D**) Gene Ontology (GO) analysis of differential genes between cluster 1 and cluster 2. (**E**) Kyoto Encyclopedia of Genes and Genomes (KEGG) analysis of differential genes between cluster 1 and cluster 2. (**F**) Heatmap displaying the hallmark pathways in different PCD clusters.

The TCGA-Advanced NSCLC cohort underwent clustering analysis based on 12 prognostic-related PCD gene expressions using the non-negative matrix factorization (NMF) algorithm. The research identified two distinct clusters (Figure 3A, 3B), with patients in cluster 1 showing significantly worse survival prognosis compared to cluster 2 (P=0.037, Figure 3C). The clinical statistical analysis results between clinical factors and two clusters are shown in Supplementary Table 2. These findings suggest that the expression patterns of these PCD genes can effectively stratify advanced NSCLC patients based on their survival outcomes. A total of 359 DEGs were identified between cluster 1 and cluster 2 (Supplementary Table 3). these DEGs were used for GO enrichment analysis, and numerous metabolic and immune-related biological processes were significantly enriched (Figure 3D). These processes included the icosanoid metabolic process, metabolic hormone process, tertiary alcohol metabolic process, antigen processing and presentation, and leukocyte-mediated cytotoxicity. The KEGG pathway enrichment analysis also demonstrated significant enrichment in 17 pathways (Figure 3E), including cytokine-cytokine receptor interaction, tight junction, arachidonic acid metabolism, and steroid hormone biosynthesis.

Moreover, substantial differences were observed in several hallmark pathways between the two clusters (Figure 3F). These included G2M checkpoint, MYC targets, oxidative phosphorylation, DNA repair, apoptosis, NOTCH signaling, and KRAS signaling. These findings shed light on the significantly predictive value of PCD genes in advanced NSCLC.

### Development of the prognosis risk model for advanced NSCLC based on the PCD-related subtypes

**Figure 4:**
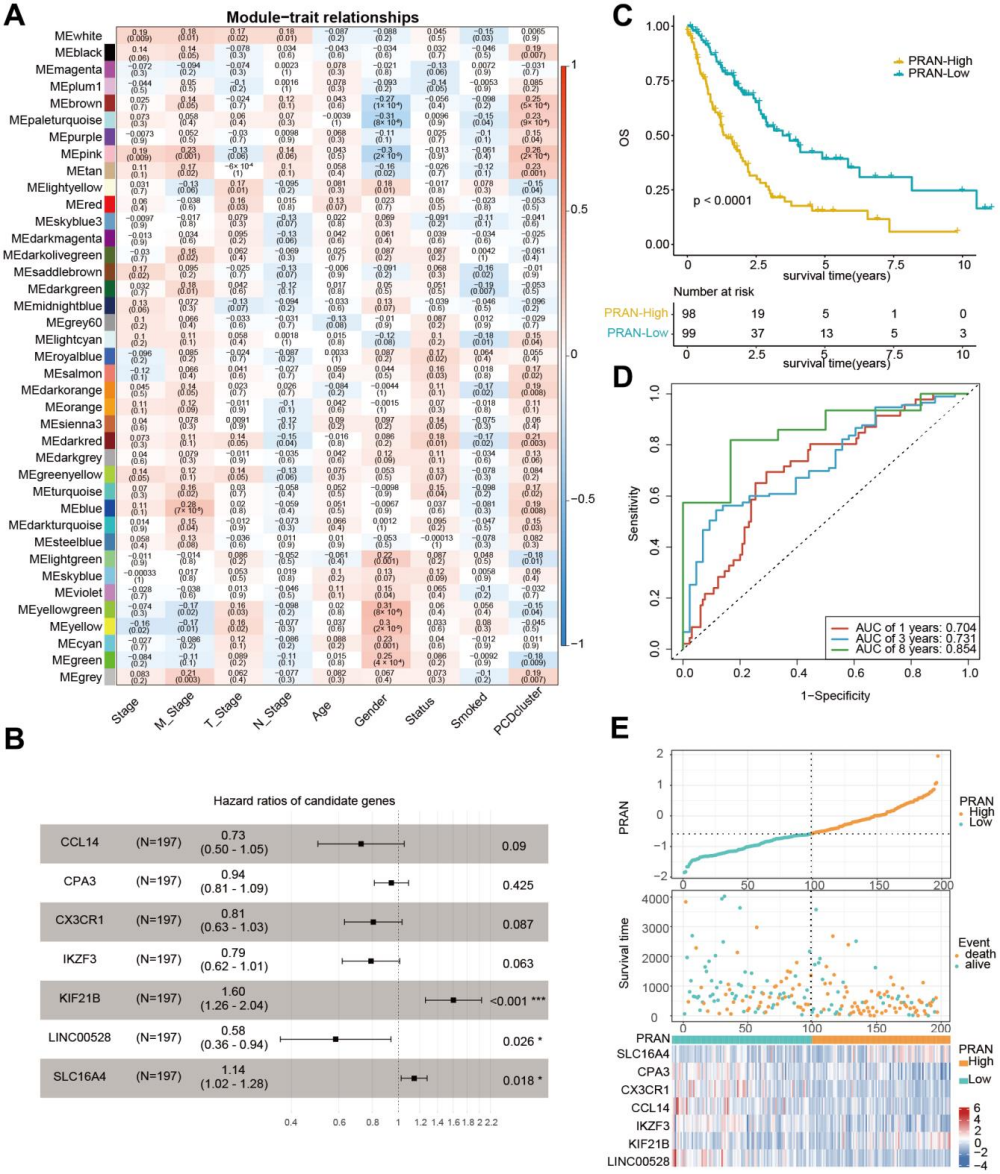
**Development of prognostic signature using PCD-related genes in TCGA-Advanced NSCLC dataset.** (**A**) Heatmap of correlation between gene modules and clinical traits, each cell containing Pearson’s correlation coefficient and p-value. (**B**) Forest plot depicting the associations between the expression levels of seven PCD genes and overall survival (OS) in the training cohort. Hazard Ratio (HR), 95% Confidence Interval (CI), and p-value were determined by multivariate Cox regression analysis. (**C**) Kaplan-Meier (KM) curve analysis of the prognostic model in the training set, showing the survival differences between high-risk and low-risk groups. (**D**) Time-dependent receiver operating characteristic (ROC) curves and area under the curve (AUC) values of the PRAN model for predicting survival status in 1-, 3-, and 8-year. (**E**) Comparison of PRAN scores, survival status, and expression of seven PCD genes between PRAN-High and PRAN-Low groups. P-value: * < 0.05; *** < 0.001.

According to WGCNA analysis, the gene co-expression network was constructed to find the critical modules associated with metastatic status and PCD cluster-related subtypes. A total of 39 modules were identified, and the “pink” (PCD cluster: R=0.26, p=2e-04, M stage: R=0.23, p=0.001), “tan” (PCD cluster: R=0.23, p=0.001, M stage: R=0.17, p=0.02), and “brown” (PCD cluster: R=0.25, p=5e-04, M stage: R=0.114, p=0.05) modules were significantly associated with PCD clusters and metastatic status (Figure 4A and Supplementary Figure 2A–2C). Next, 55 genes were initially selected through univariate Cox analysis from 1460 module-related genes (Supplementary Table 4). Subsequently, the LASSO method identified 15 genes (Supplementary Table 4 and Supplementary Figure 3A, 3B). Finally, seven genes (*CCL14*, *CPA3*, *CX3CR1*, IKAROS family zinc finger 3 (*IKZF3*), kinesin family member 21B (*KIF21B*), long intergenic non-protein coding RNA 528 (*LINC00528*), and solute carrier family 16 member 4 (*SLC16A4*) were identified based on the multivariate Cox regression analysis of all different combinations of the above 15 genes (Supplementary Table 4). The multivariate Cox regression analysis demonstrated the significant predictive value of the identified seven genes (Figure 4B). The PCD-related risk score for the Advanced NSCLC (PRAN) model was developed using RNA expression values and multivariate Cox regression coefficients of seven PCD-related genes. The formula was as follows: PRANScore=−0.32×*CCL14*-0.06×*CPA3*-0.22×*CX3CR1*-0.23×*IKZF3*+0.47×*KIF21B*-0.54×*LINC00528*+0.13×*SLC16A4*. Based on the PRAN risk model, advanced NSCLC patients were classified into two groups. The PRAN-Low group exhibited significantly better overall survival (OS) compared to the PRAN-High group (P<0.0001, Figure 4C). The area under the ROC curve (AUC) for predicting 1-, 3-, and 8-year survival using the PRAN risk model were 0.704, 0.731, and 0.854, respectively (Figure 4D). In addition, seven model genes’ expression significantly differed between PRAN-High and PRAN-Low groups (Figure 4E). These findings highlight the potential of the PRAN model as a valuable prognostic tool for predicting survival outcomes in advanced NSCLC patients.

**Figure 5:**
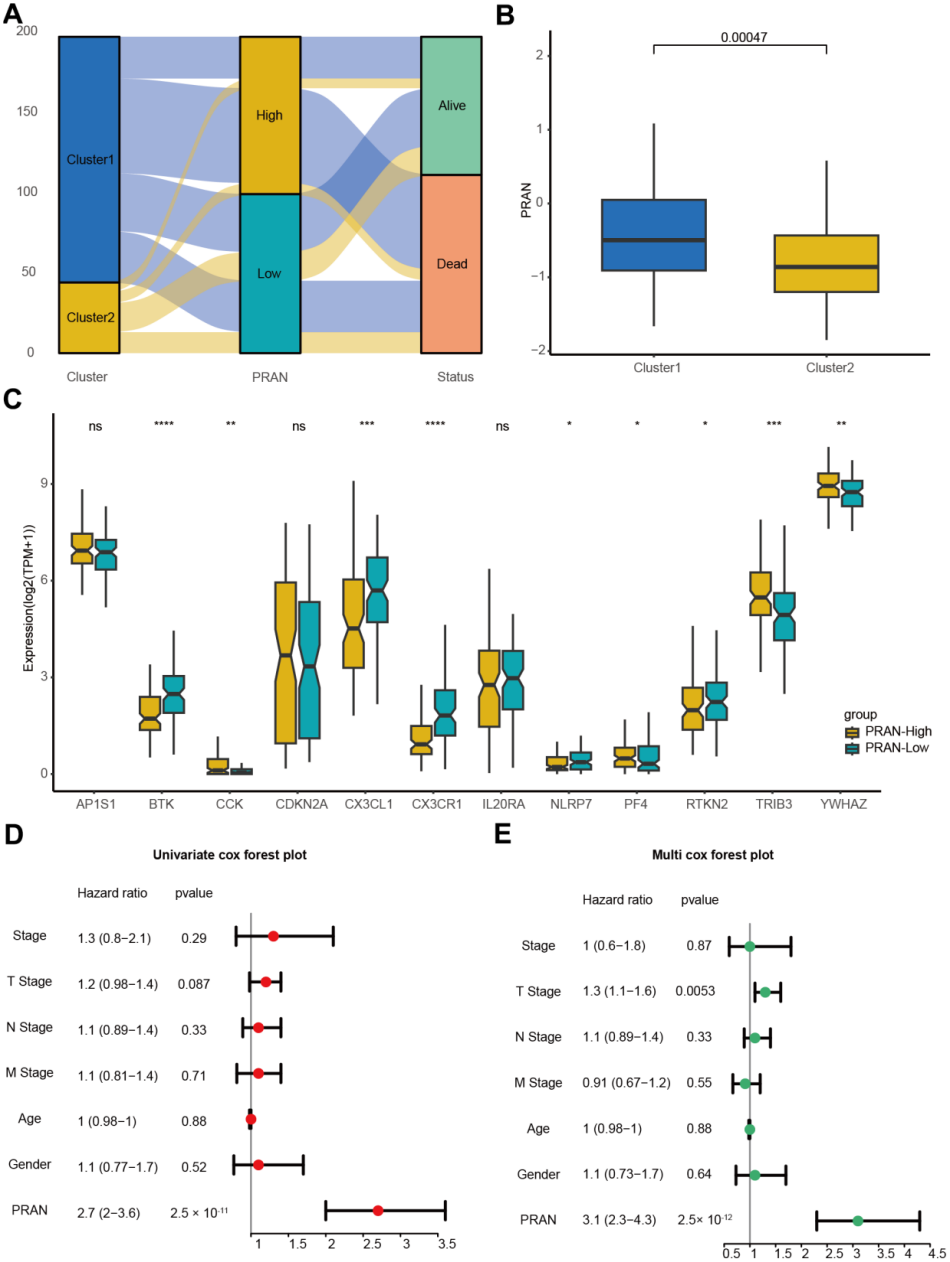
**The correlation validation of PRAN risk model in TCGA-Advanced NSCLC dataset.** (**A**) The Sankey plot illustrates the distribution of PCD risk groups, PCD clusters, and survival outcomes. (**B**) Box plots depicting the relationship between PCD clusters and PRAN risk groups. (**C**) Box plots showing the expression levels of 12 PCD-related prognosis genes between PRAN-High and PRAN-Low groups. (**D**) Univariate Cox regression analysis of PCD risk scores and clinical variables. (**E**) Multivariate Cox regression analysis of PCD risk scores and clinical variables. P-value: ns >=0.05; * < 0.05; ** < 0.01; *** < 0.001; **** < 0.0001.

The correlation analysis between the PCD subtype and the PRAN risk model revealed that the majority of patients in cluster 1 were classified into the PRAN-High group, and the PRAN score exhibited a significantly higher value in cluster 1 compared to cluster 2, indicating an increased risk in advanced NSCLC (P=0.00047, Figure 5A, 5B). Upon further investigating the correlation between the previously identified 12 PCR-related genes and the PRAN risk model, we observed significant expression differences between the PRAN-High and PRAN-Low groups (Figure 5C). Through comprehensive univariate (Figure 5D) and multivariate Cox (Figure 5E) regression analyses, we systematically investigated the prognostic significance of both clinical characteristics (stage, age, and gender) and the PRAN risk model in advanced NSCLC. Univariate Cox regression indicated PRAN was a risk factor for the overall survival in advanced NSCLC (P=2.5e-11, HR=2.7, 95% CI =2-3.6). Meanwhile, Multivariate Cox regression analysis further confirmed the prognosis significance of PRAN in advanced NSCLC (P=2.5e-12, HR=3.1, 95% CI=2.3-4.3). Our findings demonstrated that the model exhibited remarkable stability and superior predictive value compared with clinical factors.

Somatic mutation profile analysis revealed that mutations of filaggrin (*FLG*) and CUB and Sushi multiple domains 1 (*CSMD1*) frequently occurred in the PRAN-High group, while zinc finger homeobox 4 (*ZFHX4*) and xin actin binding repeat containing 2 (*XIRP2*) mutated more in the PRAN-Low group (Supplementary Figure 3C). TMB different analysis demonstrates no difference between PRAN-High and PRAN-Low groups (Supplementary Figure 3D). However, the PRAN-Low group showed higher IFN-γ response (Supplementary Figure 3E) and TCR diversity (Supplementary Figure 3F, 3G), implying robust anti-tumor activity.

These results validate the robustness and clinical utility of the model as a reliable predictive tool for advanced NSCLC.

### Enrichment analysis and drug sensitivity prediction based on PRAN risk model for advanced NSCLC

**Figure 6:**
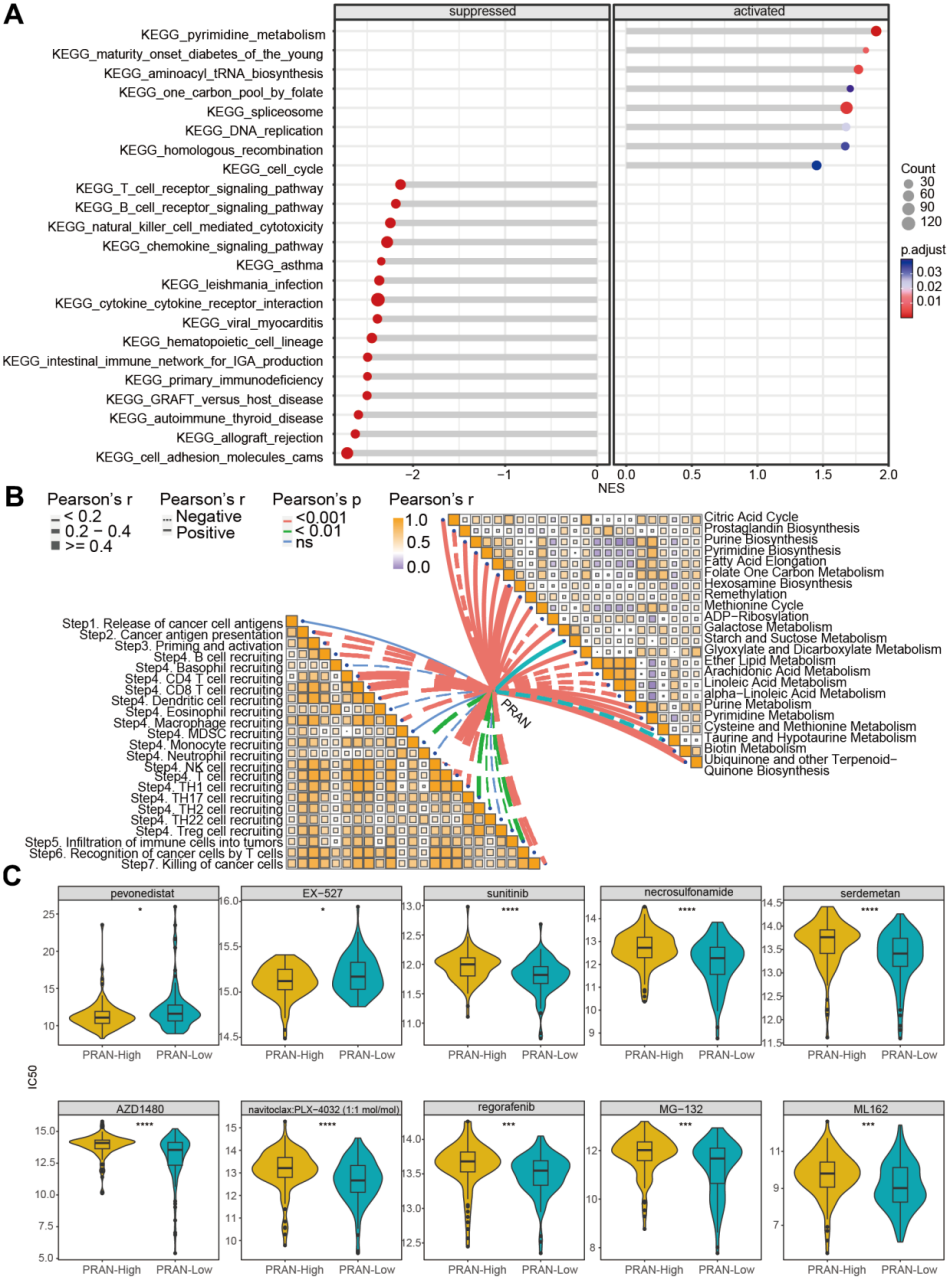
**Biological function analysis and drug-susceptibility analysis of PRAN risk model in TCGA-Advanced NSCLC dataset.** (**A**) The lollipop plot displays the top 10 significantly enriched suppressed pathways and all activated pathways in the PRAN-High and PRAN-Low groups. (**B**) Pearson correlation analysis demonstrates the relationship between PCD scores and cancer immune cycle activity (left) and metabolism-related pathways (right). (**C**) Sensitivity analysis of anti-tumor drugs between PRAN-High and PRAN-Low groups. P-value: ns >=0.05; * < 0.05; ** < 0.01; *** < 0.001; **** < 0.0001.

To further explore the enrichment of molecular biological processes and signaling pathways in the PRAN-High and PRAN-Low groups, we conducted a GSEA analysis. The results revealed significant enrichment of pyrimidine metabolism, spliceosome, and aminoacyl tRNA biosynthesis pathway in the PRAN-High group. In contrast, cell adhesion molecules cams, cytokine-cytokine receptor interaction, T/B cell receptor signaling, cell cycle pathway, and so on, in the PRAN-Low group (Figure 6A).

Next, we analyzed the correlation of PRAN score with cancer immune cycle activity and metabolism-related pathways (Figure 6B). Most immune cycle activity was negatively related to the PRAN score, implying decreased anti-tumor immunity in the higher PRAN score population. Interestingly, diverse correlations with the PRAN score were observed concerning metabolic pathways. Notably, the Hexosamine biosynthesis pathway demonstrated the strongest positive correlation with the PRAN score, highlighting its critical role in the PRAN-High group. Additionally, several signaling pathways, including Galactose metabolism, Glyoxylate and Dicarboxylate metabolism, Purine metabolism, Pyrimidine metabolism, Cysteine and methionine metabolism, Biotin metabolism, and Ubiquinone and other terpenoid-Quinone biosynthesis were significantly positively associated with the PRAN score. Conversely, ADP-Ribosylation, Arachidonic acid metabolism, linoleic acid metabolism, and alpha-linoleic acid metabolism exhibited significant negative correlations with the PRAN score. These findings provide valuable insights into the complex interplay between the PRAN score and metabolic pathways, contributing to a deeper understanding of the metabolic dysregulation underlying advanced NSCLC.

The results above indicate a significant correlation between the PRAN score, immune microenvironment activity, and metabolic processes in advanced NSCLC. Furthermore, we predicted the potential drug sensitivities for patients in different PRAN risk groups (Figure 6C). The complete sensitive drugs and statistical data are presented in Supplementary Table 5. The smaller IC_50_ value indicates that inhibitors function more effectively with targeted compounds [19]. Remarkably, the PRAN-Low group demonstrated notable sensitivity to sunitinib, AZD1480, regorafenib, and five PCD-targeted drugs, including necrosulfonamide (necrosis apoptosis inhibitor), Serdemetan (HDM2 inhibitor, delayed apoptosis), navitoclax: PLX-4032 (target BCL2 and BRAF, apoptosis inhibitor), MG-132 (inducing apoptosis, activating autophagy), and ML162 (inducing ferroptosis), whereas the PRAN-High group showed significant sensitivity to pevonedistat (causing cell death) and EX-527 (enhancing autophagy). These results underscore the clinical implications of the PRAN model in guiding personalized treatment strategies in advanced NSCLC patients.

**Figure 7:**
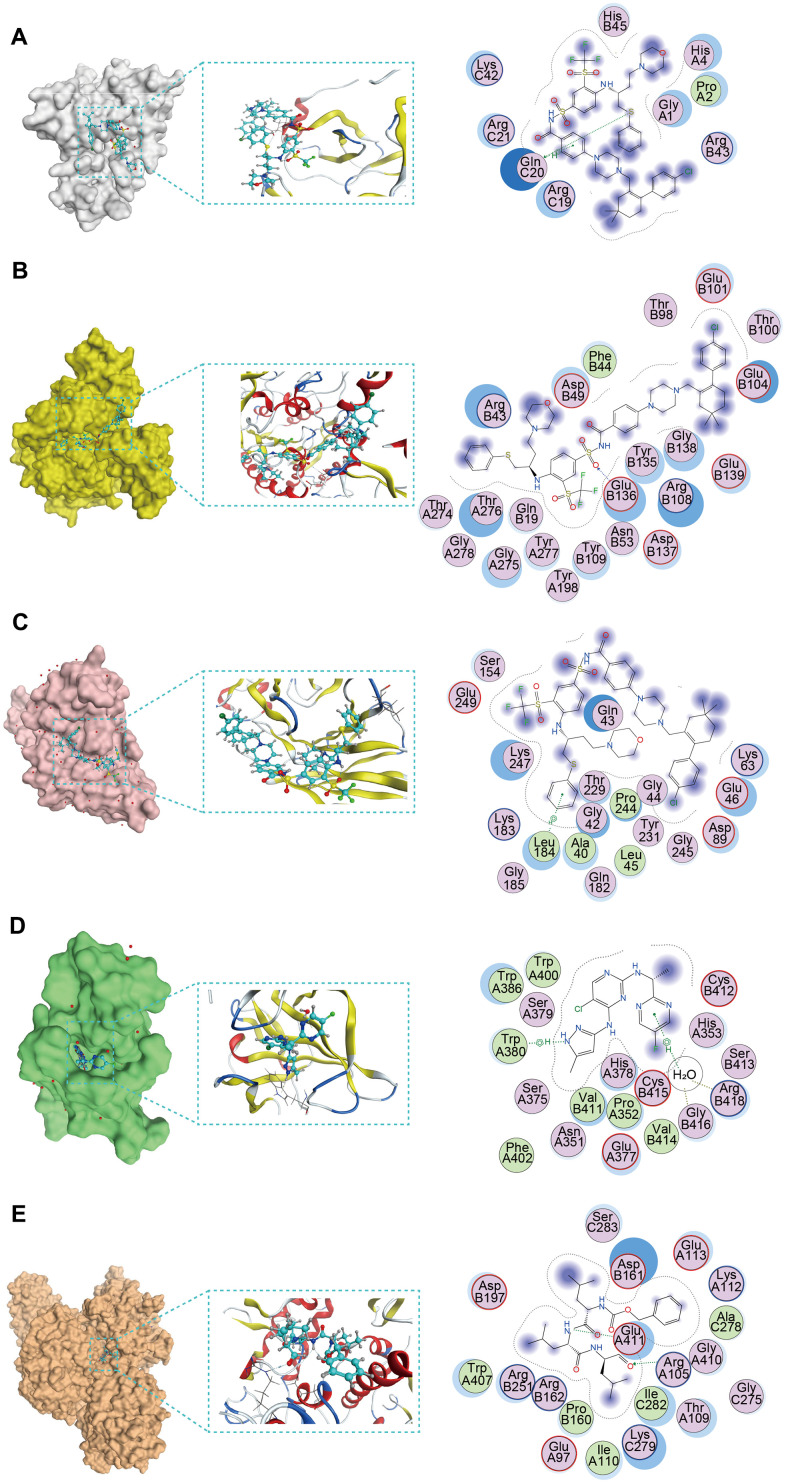
**The molecular docking posture predicting for the sensitive anti-tumor drugs and targeted PCD genes.** (**A**) Docking position of *CCL14* active pocket with navitoclax. (**B**) Docking position of *CPA3* active pocket with navitoclax. (**C**) Docking position of *CX3CR1* with navitoclax. (**D**) Docking position of *IKZF3* with AZD1480. (**E**) Docking position of *KIF21B* with MG-132.

Molecular docking was used to screen potential drug candidates and elucidate the molecular mechanisms involved. We employed MOE software to simulate the binding modes of small molecule drugs to seven PCD-related genes**.** As revealed by molecular docking analyses, CCL14, CPA3, CX3CR1, IKZF3, and KIF21B demonstrated the potential to interact with these small molecule drugs (Supplementary Table 6). Furthermore, based on these analyses, we identified the top-ranked drug for each target protein with the highest binding affinity Figure 7A–7E). The navitoclax closely bonds with the CCL14 protein by the amino acid residue of Gln-C20 (Figure 7A). The navitoclax closely bonds with the CPA3 protein by the amino acid residues of Glu-B136 (Figure 7B). The navitoclax closely bonds with CX3CR1 protein by the amino acid residue of Leu-184 (Figure 7C). The AZD1480 closely bonds with the IKZF3 protein by the amino acid residues of Arg-B418, Gly-B416, and Trp-A380 (Figure 7D). The MG-132 closely bonds with the KIF21B protein by the amino acid residue of Glu-A411 and Arg-A105 (Figure 7E).

### Tumor immune microenvironment (TIME) characteristics assessment based on the PRAN risk model for advanced NSCLC

**Figure 8:**
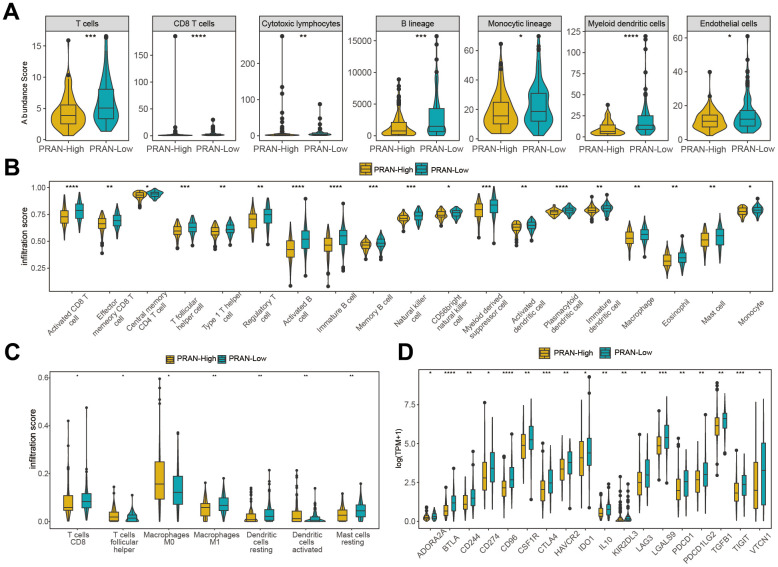
**Immune infiltration analysis of PRAN-High and PRAN-Low groups TCGA-Advanced NSCLC dataset.** (**A**) Violin plot showing the immune cell enrichment scores of PRAN-High and PRAN-Low groups, assessed using MCPcounter. (**B**) The ssGSEA algorithm shows the immune cell infiltration of immune-related functions and pathways in PRAN-High and PRAN-Low groups. (**C**) Immune cell infiltration content of PRAN-High and PRAN-Low groups was analyzed using the CIBERSORT algorithm. The analysis provides insights into the proportion of different immune cell types in each risk group. (**D**) Expression of immunoinhibitor genes between PRAN-High and PRAN-Low groups. P-value: * < 0.05; ** < 0.01; *** < 0.001; **** < 0.0001.

We utilized various algorithms to analyze immune cell abundance across different PRAN subgroups in the TCGA-Advanced NSCLC dataset. The MCP counter analysis demonstrated significantly higher enrichment scores of T cells, B lineage cells, monocytic lineage cells, myeloid dendritic cells, and endothelial cells in the PRAN-Low group (Figure 8A). Consistently, ssGSEA analysis revealed enhanced immune cell infiltration in the PRAN-Low group, corroborating the MCP counter findings (Figure 8B). Furthermore, CIBERSORT algorithm results confirmed a higher infiltration content of CD8 T cells and macrophages M1 cells in the PRAN-Low group, further supporting the robust anti-tumor immune activity associated with the PRAN-Low group. Interestingly, the PRAN-High group exhibited significantly higher infiltration content of macrophages M0, T cell follicular helper cells, and activated dendritic cells, suggesting that the survival advantage conferred by these three immune cell populations may be offset by other factors within the TIME (Figure 8C). The PRAN-High group exhibited significantly lower ESTIMATE scores, as well as lower stromal scores and immune scores, indicating higher tumor purity and increased infiltration of tumor cells (Supplementary Figure 4A). The expression of HLA (Supplementary Figure 4B) and immune inhibitors genes (Supplementary Figure 4C) were higher in the PRAN-Low group compared with the PRAN-High group. At the same time, the expression of immunostimulatory genes was also significantly upregulated against the antigen presentation and immune response (Figure 8D). These findings provide comprehensive insights into the intricate interplay between TIME characteristics and the PRAN model, underscoring the complex dynamics of the tumor immune microenvironment in advanced NSCLC.

### Validation of the PRAN risk model for advanced NSCLC

**Figure 9:**
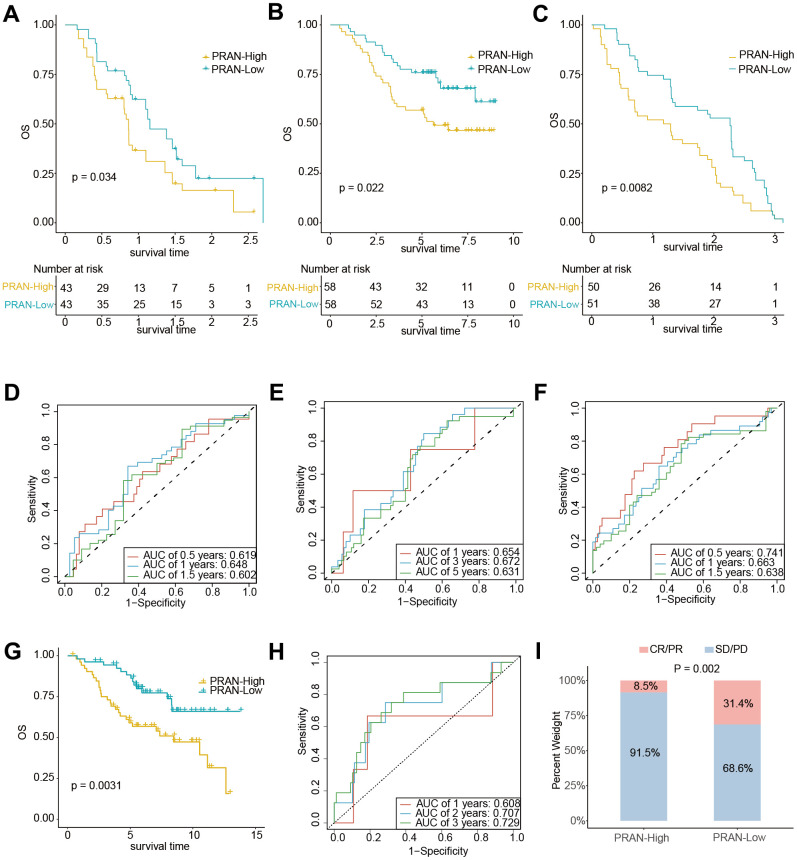
**The predicting performance validation of the PRAN risk model in multiple GEO cohorts.** (**A**–**C**) Kaplan-Meier survival analysis in NSCLC validation cohort GSE61676, GSE13213, and GSE91061. (**D**–**F**) Time-dependent ROC curves between PRAN-High and PRAN-Low groups in validation cohort GSE61676, GSE13213, and GSE377453. (**G**) Kaplan-Meier survival analysis in validation cohort GSE74777. (**H**) Time-dependent ROC curves between PRAN-High and PRAN-Low groups in validation cohort GSE74777. (**I**) The predicting performance of immunotherapeutic efficiency of PRAN risk model in the GSE91061 cohort.

The extensive analyses performed in this study provide compelling evidence supporting the PRAN risk model’s ability to accurately predict survival prognosis and TIME characteristics of patients with advanced NSCLC. We conducted rigorous validation across multiple independent validation cohorts to further validate the predicted performance. Survival analysis conducted in two independent NSCLC validation cohorts, namely GSE61676 (P=0.034, Figure 9A) and GSE13213 (P=0.022, Figure 9B), revealed a significant difference in overall survival (OS) between the PRAN-High and PRAN-Low groups. The respective optimal AUC values for predicting performance were 0.648 (Figure 9D) and 0.672 (Figure 9E). These results further validate the predictive utility of the PRAN model in accurately stratifying patients with advanced NSCLC.

What’s more, consistent with the analysis results of the advanced NSCLC dataset, the patients with early NSCLC from the GSE74777 and GSE50081cohort and those in the PRAN-High showed worse prognoses than those in the PRAN-Low (Figure 9G and Supplementary Figure 5A). In addition, the predicted survival ROC curve confirmed the precise predictive capacity of the risk model, with area under the ROC curve (AUC) values of 0.608, 0.707, 0.729 and 0.585, 0.599, 0.603 for 1-, 2-, and 3-year survival, respectively (Figure 9H and Supplementary Figure 5B).

We sought to assess the PRAN risk model’s predictive capacity in determining patients’ immunotherapy responses. Survival analyses were performed in two immunotherapy cohorts of lung cancer (GSE135222 and GSE93157) and one melanoma (GSE91061). Due to the limited sample size in the GSE135222 (Supplementary Figure 5C) and GSE93157 (Supplementary Figure 5D) cohorts, we did not observe a significant difference in PFS between the two PRAN subgroups. However, we observed a slightly better prognosis in the PRAN-Low group than in the PRAN-High group. In contrast, in the independent melanoma immunotherapy cohort GSE91061, the PRAN-Low group demonstrated a significantly superior OS benefit compared to the PRAN-High group (P=0.0082, Figure 9C), with an optimal AUC of 0.741 (Figure 9F) for predicting performance. Notably, the PRAN-High group exhibited a significantly lower immunotherapy response rate than the PRAN-Low group in the GSE91061 NSCLC cohort (P=0.002, Figure 9I). These findings further reinforced the value of PRAN model as a predictive tool in guiding treatment decisions for patients with advanced NSCLC.

### Establishment of a nomogram based on the PRAN risk model and clinical factors for advanced NSCLC

**Figure 10:**
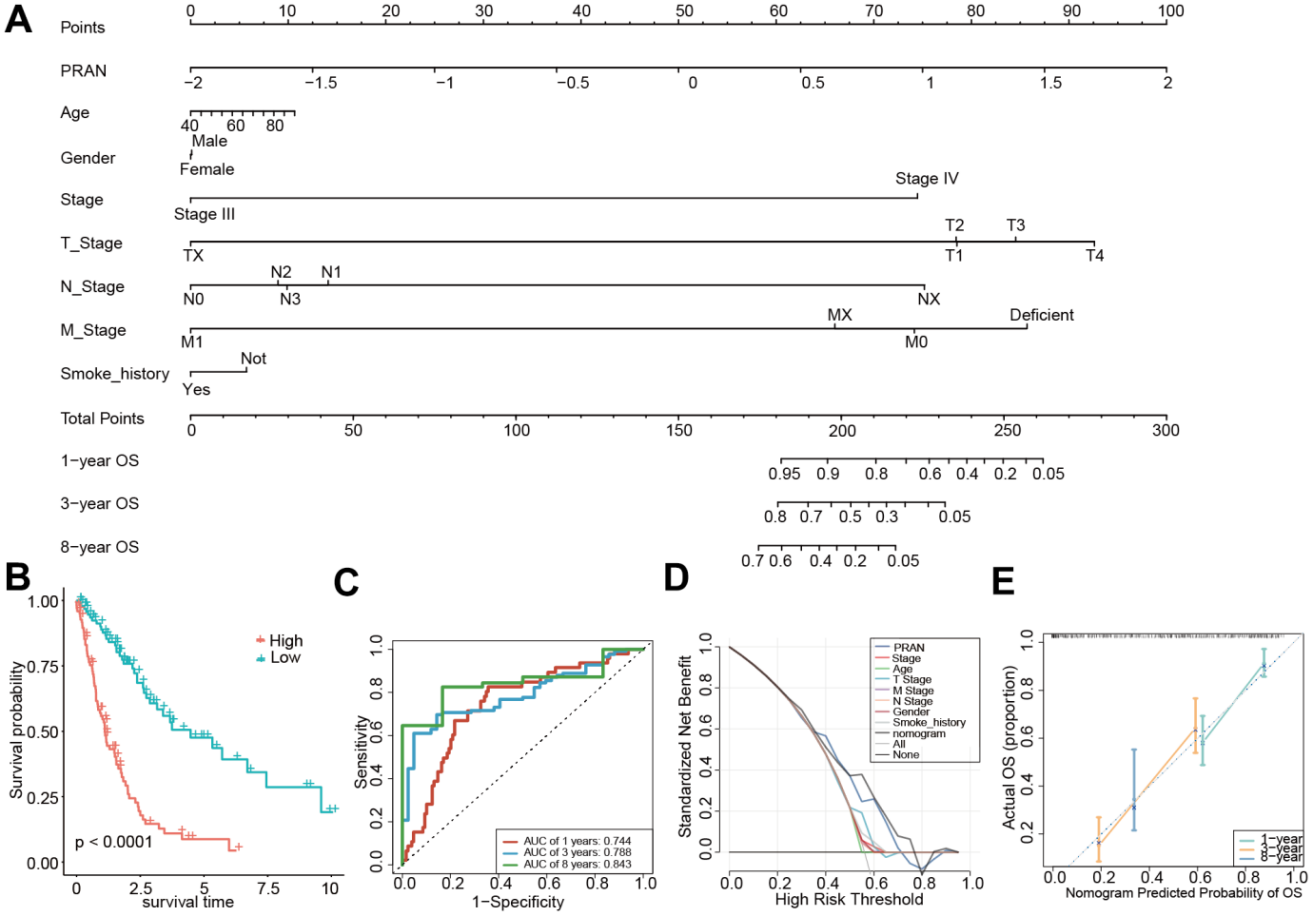
**Construction and validation of the PRAN score-based nomogram.** (**A**) The nomogram plot was constructed in the training cohort with incorporation of PRAN and clinical characteristics. (**B**) Kaplan-Meier survival curves based on PRAN scores calculated using the nomogram. (**C**) ROC curves for predicting 1-year, 3-year and 8-year OS for the nomogram. (**D**) Decision curve analysis of nomogram, PRAN risk model and clinical characteristics. The black line in this Figure indicates the assumption of no patient death. (**E**) Nomogram calibration plot based on the agreement between predicted and observed values at 1, 3, and 8 years. X-axis is nomogram predicted overall survival, y-axis is actual overall survival, dashed line is ideal performance of nomogram, and 95% confidence interval is represented by closed vertical line.

Finally, for better application in clinical practice, we established a nomogram based on the multivariate Cox regression analysis of the PRAN score, and age, stage, smoking status, and gender were included (Figure 10A). Survival analysis demonstrated a significant difference in OS between the two subgroups based on the nomogram score, with the high-risk group exhibiting worse OS outcomes (P<0.0001, Figure 10B). The nomogram showed an optimal AUC value of 0.843, indicating its predictive solid performance (Figure 10C). Furthermore, DCA results confirmed the nomogram’s robustness and optimal predictive ability (Figure 10D). Calibration curves were generated to assess the accuracy of the nomogram in predicting the 1-, 3-, and 8-year survival rates, further validating its predictive accuracy (Figure 10E). The nomogram could be a practical tool for prognostic assessment and aid in clinical decision-making.

### The mRNA and protein expression difference of seven PRAN-genes between tumor and normal cells in NSCLC

**Figure 11:**
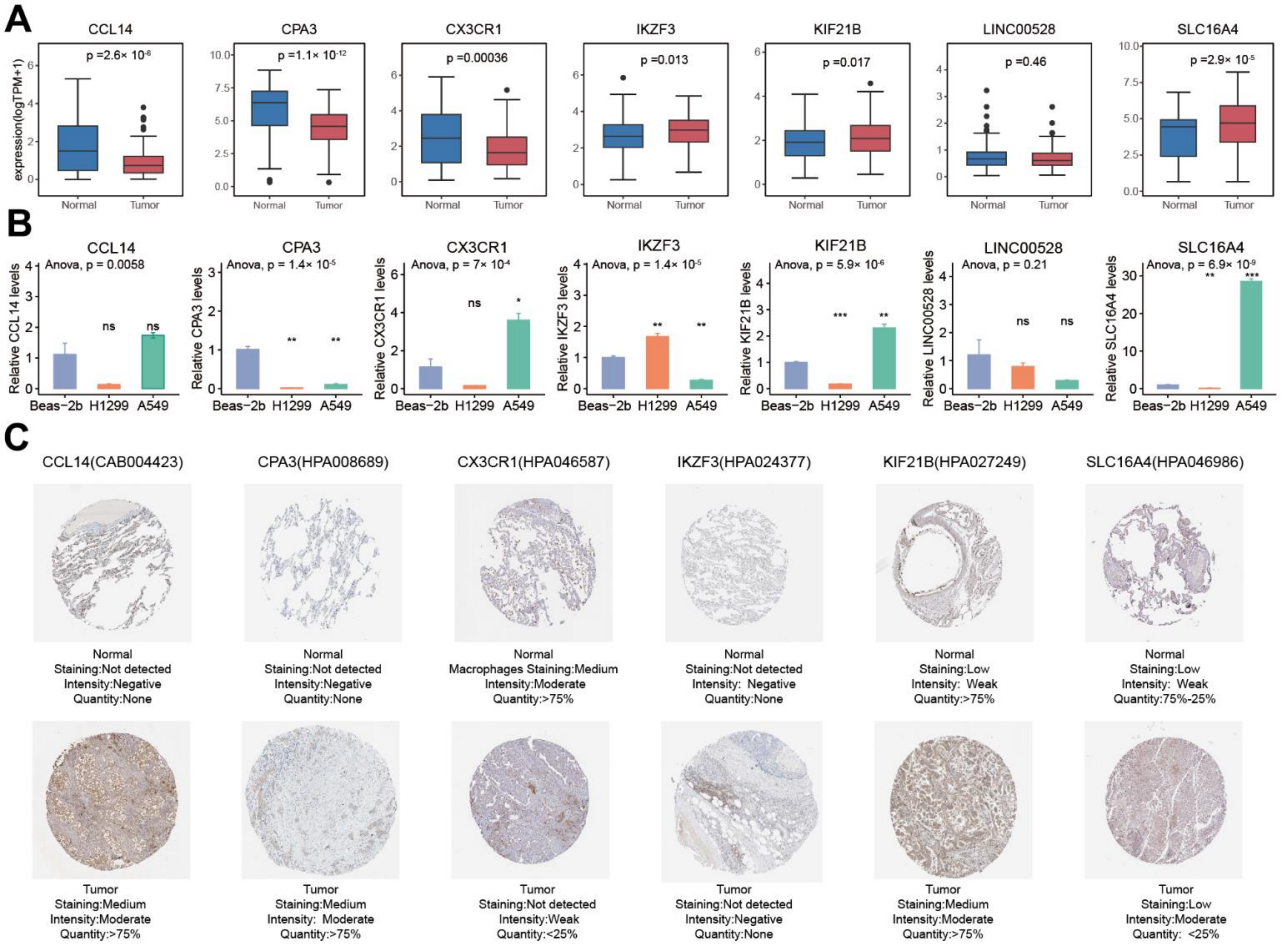
**Comparison analysis of the expression of seven PRAN model genes between NSCLC tumor and normal samples at RNA and protein levels.** (**A**) RNA expression differences of seven PRAN model genes between tumor and normal samples in TCGA-Advanced NSCLC. (**B**) RNA expression differences of seven PRAN model genes between two tumor cell lines and one normal cell line. (**C**) The immunohistochemistry image of CCL4 (CAB004423), CPA3 (HPA008689), CX3CR1 (HPA046587), IKZF3 (HPA024377), KIF21B (HPA027249), and SLC16A4 (HPA046986) from HPA database. The URLs of the source of each image were shown in Supplementary Table 7.

The mRNA expression of seven PRAN genes (*CCL14, CPA3, CX3CR1, IKZF3, KIF21B, LINC00528*, and *SLC16A4*) exhibited noticeable difference between normal and tumor in NSCLC (Figure 11A). According to the RNA expression validation in two human NSCLC cell lines, A549 and NCI-H1299, and one human lung bronchial epithelial cell line (BEAS-2B), significant expression differences were observed between normal and tumor cells (Figure 11B). H1299 was a p53 deficient cell line, and the expression of *CCL14, CPA3, CX3CR1, KIF21B*, and *SLC16A4* was lower than in A549 and Beas-2b. The expression of *IKZF3* was higher in H1299 cells than in A549 and Beas-2b. The expression of *IKZF3, CPA3*, and *LINC00528* were down-regulated in A549 cells than in normal cells, while the expression of *CCL14, CX3CR1, KIF21B*, and *SLC16A4* were up-regulated in A549 cell than in normal cell. The protein expression results acquired from the Human Protein Atlas (HPA) database were consistent with the mRNA results, showing the significantly different between NSCLC tumor and normal tissues (Figure 11C and Supplementary Table 7).

## DISCUSSION

As a common cancer with high morbidity and mortality, lung cancer still needs excellent attention [20]. Advanced NSCLC poses treatment challenges and a bleak prognosis, especially patients with metastatic NSCLC often experience low survival rates [21]. In recent years, ICIs have garnered widespread utilization in lung cancer treatment, particularly advanced metastatic NSCLC, yielding promising outcomes [22, 23]. Hence, investigating molecular markers associated with NSCLC prognosis and immunotherapy is imperative to inform treatment decisions for advanced NSCLC patients. Multiple studies support the use of risk models to predict tumor immunotherapy responses and guide personalized medicine [24–26].

We rigorously examined the prognostic relevance of tumor programmed cell death processes in advanced NSCLC. We identified twelve DEGs, all showing significant predictive value in advanced NSCLC. According to RNA expression of these twelve genes, molecular subtyping analysis identified two distinct subtypes with divergent survival outcomes. Among them, *CDKN2A* exhibited the highest mutation frequency and CNV deletion. *CDKN2A*, as a cell cycle regulation gene, is associated with cuproptosis processes and is shown to impact cancer patients’ prognosis [27].

Through a comprehensive approach, our study aimed to gain a profound understanding of the intricate relationship between the identified genes and the specific risk associated with advanced NSCLC. The mRNA expression of seven PRAN genes (*CCL14, CPA3, CX3CR1, IKZF3, KIF21B, LINC00528*, and *SLC16A4*) showed significant differences between normal and tumor samples in the advanced NSCLC cohort. The immunohistochemistry (IHC) stained images obtained from the HPA dataset also demonstrate differential protein expression. These findings were further validated through RT-qPCR experiments in A549 and NCI-H1299 (p53 deficient), and BEAS-2B cell lines, consistently showing significant differences in gene expression between normal and tumor cells. However, the expression of these genes in A549 and H1299 cells exhibited some inconsistency, potentially due to the underlying heterogeneity of NSCLC tumors, as A549 and H1299 represent different subtypes with distinct genomic aberrations [28]. We speculate that this difference may arise from specific molecular mechanisms of cell lines, such as cell line origin, differentiation status, gene mutation, epigenetic regulation, and heterogeneity of transcription factors. In addition, A549 and H1299 are cell lines of adenocarcinoma and large cell carcinoma respectively, and they also have significant differences in cell morphology and molecular characteristics. This difference may affect the activation or inhibition of intracellular signaling pathways, resulting in differences in gene expression levels.

The PRAN-High group is associated with pyrimidine, spliceosome processes, and various metabolic pathways (e.g., hexosamine biosynthesis) based on biological function and signaling pathway enrichment. Pyrimidine synthesis enhances Notch signaling and upregulates c-Myc expression at the transcriptional level, leading to an increase in key glycolytic enzymes. The key enzymes involved in up-regulating pyrimidine synthesis, CAD and DHODH, are implicated in enhancing the chemoresistance of gastric cancer by accelerating glycolysis. Conversely, they sensitize cancer cells to chemotherapy *in vitro* and *in vivo* by inhibiting the pyrimidine biosynthesis pathway [29]. Pyrimidine synthesis is demonstrated up-regulation in Glioblastoma stem cells (GSCs) and is associated with poor survival in glioma patients [30]. The spliceosome components and splicing factor play an essential role in cancers. Notably, splicing alterations can profoundly affect downstream cellular signaling, contributing to cancer development and progression [31]. Metabolic pathways have established crucial roles in cancer development and proliferation over the decades. Moreover, it has become increasingly evident that metabolic pathways closely interact with immune cells during reprogramming cancer cell metabolism [9, 32]. The hexosamine biosynthesis pathway, a vital part of glucose metabolism, is consistently enhanced in cancers during progression [33]. These remarkable insights into the intricate connections between metabolism pathways and cancer biology have revealed the underlying biological mechanism of the PRAN risk model.

Interestingly, the PRAN-High group observed significant enrichment of T-cell follicular helper cells and activated dendritic cells. The T follicular helper cell is related to better survival and therapeutic outcomes in NSCLC, which may be because the T follicular helper cell serves as a critical physiological source of IL-21 for CD8 T cell infiltrating the tumor [34]. Activated dendritic cells serve as an antigen presentation vector, and the related neoantigen vaccine has become an effective therapeutic drug that could improve the survival status of patients with advanced lung cancer [35]. When immune-activating and immune-stimulating cells coexist, their mutual interactions finally have a poor impact on the survival outcomes of patients in the PRAN-High group, and the survival advantage may be offset [36].

To facilitate the practical application of the PRAN risk model in clinical practice, we have combined the model with patients’ baseline characteristics to construct a more intuitive nomogram. Its superior performance enhances clinical transformation and aids in decision-making for therapeutic strategies.

Furthermore, we performed sensitive drug prediction on the PRAN risk model. Notably, seven of these drugs specifically target the cell death pathway, leveraging the regulatory role of cell death in tumor biology processes to improve patient survival outcomes. Necrosulfonamide, as an inhibitor of necroptosis, exerts its action by targeting mixed lineage kinase domain-like protein (MLKL), which is pivotal in initiating necroptosis and triggering apoptosis in cancer cells [37]. Ferris et al. uncovered the mechanism of pevonedistat in inducting cell death for colorectal cancer (CRC) therapy, and the responses were effective for both p53 wild-type and mutant mCRC, implying the applicable potent of pevonedistat in other cancers [38]. EX-527 could effectively reverse the AICAR-induced downregulation of c-Myc and metadherin (*MTDH*) expression. Moreover, without *MTDH*, treatment with EX-527 will significantly increase breast cancer cell death [39]. Collectively, these studies offer compelling evidence and elucidate the molecular mechanisms associated with cell death inhibitors, suggesting their promising potential in advanced NSCLC therapy. Moreover, molecule docking analysis further explores the optimal binding position between target PRAN genes and small molecule drugs. As a BCL-2 inhibitor, the navitoclax closely binds with proteins and amino acid residues of hub genes *CCL14, CPA3*, and *CX3CR1*. Having been demonstrated synergy therapeutic efficient when combined with targeted therapies in hematologic malignancies, the navitoclax exhibited compelling clinical efficacy when combined with Osimertinib for *EGFR*-mutant NSCLC patients in a phase IB clinical study (NCT02520778) [40]. In addition, navitoclax also shows increased efficacy in NSCLC patients with a poor response to taxane chemotherapy [41]. These findings shed new light and a solid foundation for the clinical therapy application of navitoclax.

There is still a limitation in our study. We performed the validation experiment using NSCLC cell lines, which cannot reveal the heterogeneous characteristics and prognosis significance underlying advanced NSCLC tumor cells. Further studies across paired advanced NSCLC and normal tissues are warranted to fully elucidate the heterogeneity and characterize subtype-specific expression profiles related to NSCLC pathogenesis.

In conclusion, our findings contribute to advancing precision medicine approaches in NSCLC management. The identified prognostic and immunotherapy biomarkers offer promising avenues for tailoring treatment strategies, optimizing patient outcomes, and enhancing the overall quality of life for those afflicted with advanced NSCLC. As we continue to uncover the complex molecular landscape of this disease, our predictive model presents a valuable tool to guide clinical decision-making and improve patient care. Further prospective studies are warranted to realize this model’s potential in real-world clinical practice fully.

## MATERIALS AND METHODS

### Data collection and processing

The expression data, somatic mutation information, and copy number variation (CNV) profiles of TCGA-LUAD and TCGA-LUSC from the TCGA database were downloaded from UCSC Xena (https://xenabrowser.net/datapages/), and the corresponding clinical information data were obtained using the R package “TCGAbiolinks”. One hundred ninety-seven advanced tumor samples were selected as the training set and named TCGA-Advanced NSCLC (selection criteria for advanced patients: Stage III-IV) (Supplementary Table 2). Three public datasets (GSE13213, GSE61676, GSE91061, GSE74777 and GSE50081) with available overall survival (OS) data and two datasets (GSE135222 and GSE93157) with progression-free survival (PFS) data were downloaded from the GEO database as validation sets. GSE61676 is advanced NSCLC dataset without immunotherapy, GSE13213 is NSCLC dataset without immunotherapy, GSE74777 is early LUSC dataset without immunotherapy, GSE50081 is early NSCLC dataset without immunotherapy, GSE91061 is melanoma dataset treated with anti-CTLA4 and PD-1, GSE135222 is advanced NSCLC dataset treated with anti-PD-1/PD-L1, and GSE93157 is lung cancer dataset treated with anti-PD-1. All included GEO cohorts’ sample information is presented in Supplementary Table 8.

### Identification of the expression and variation levels of PCD-related genes

We collected raw transcriptome data from 197 TCGA-Advanced NSCLC patients and 108 normal tissues from the TCGA-LUAD and TCGA-LUSC cohorts. The “limma” package was utilized to identify differentially expressed genes (DEGs) following the criteria of P adjust. < 0.05 and | log2FC| > 1. Kaplan-Meier analysis was performed to assess the impact of each DEG on overall survival (OS) time. The “maftools” package explored somatic mutations among TCGA-Advanced NSCLC patients. We visualized the distinct characteristics of PCD-related genes using a circos plot created with the “RCircos” R package.

### Unsupervised clustering and functional analysis

To explore the prognosis significance of PCD pathways, we performed the consensus unsupervised clustering analysis using the NMF package in R based on twelve PCD-related DEGs. The number of clusters k was set from 2 to 10. The stability of clusters obtained through NMF was assessed by the cophenetic correlation, which ranged from 0 and 1. A higher value indicated more excellent cluster stability. DEGs between different PCD clusters were identified using the “limma” R package with cut-off criteria of |logFC| > 1 and P adjust. < 0.05. The “clusterProfiler” R package was used to perform enrichment analysis on the DEGs.

### Selection of modules associated with PCD clusters traits by WGCNA

We performed Weighted Gene Co-expression Network Analysis (WGCNA) using the R package “WGCNA” to identify PCD cluster-related genes based on the TPM data. We evaluated different soft thresholds (β values from 1 to 20) for scale independence and average connectivity to construct a scale-free network. The appropriate β value was selected when the scale independence exceeded 0.9 and the average connectivity remained relatively low. Next, we categorized the genes into distinct modules by calculating the topological overlap matrix (TOM). Due to metastatic invariably occurring as a late event in tumor progression and the complex relationship between metastasis and cell death, some tumor cells may increase their ability of survival and migration by inhibiting apoptosis or inducing abnormal cell death in the process of metastasis [42]. And once the tumor cells successfully metastasized to other parts, some cells may suffer cell death due to the influence of heterogeneous environment [43, 44]. Correlations between modules and clinical characteristics were assessed, and modules significantly associated with metastatic status and PCD cluster were further analyzed (p < 0.05).

### Development and validation of the risk model

Univariate Cox regression analysis was used to evaluate the prognosis value of the critical module genes and screen predictive genes (p < 0.05). The Least Absolute Shrinkage and Selection Operator (LASSO) regression analysis was performed on candidate genes significantly associated with prognosis. Then, multivariate Cox regression was conducted on all combinations of 15 candidate genes screened by LASSO analysis in the TCGA Advanced NSCLC. The risk scores selected gene combinations were calculated using the multivariate Cox regression coefficients. Finally, we constructed a predictive model with the highest accuracy and calculated risk scores as follows:


$$\text{Risk score}=\sum_{\text{i}=1}^{\text{m}}{\text{Expression}}_{\text{i}}\times {\text{Coefficient}}_{\text{i}}$$

The m is the number of signature genes for constructing the model; the “Expression_i_^”^ indicates the expression value of signature gene in the sample; the Coefficient is the multivariate Cox regression coefficient of gene.

The median risk score was used as cutoff value for patients’ classification. The receiver operating characteristic (ROC) curve was used to test the accuracy of the risk model. Therefore, the “pROC” package was applied to perform ROC analysis and calculate the area under the ROC curve (AUC) to evaluate the predictive performance of the PCD-related prognostic model. ROC curves for risk scores at 1, 3, and 8 years were plotted using the R package “timeROC”.

We constructed a nomogram prediction model using the “RMS” package, incorporating the PCD risk score and clinical factors. Calibration and ROC curves were used to evaluate the prediction accuracy of the nomogram. Additionally, we conducted a decision curve analysis (DCA) to test the nomogram’s accuracy.

### Correlation analysis between clinical characteristics, mutation, and immune-related characteristics with the PCD risk model

We performed hazard rate analysis on clinicopathological characteristics, including age, gender, stage, TNM stage, and the PCD risk score using univariate and multivariate Cox regression analyses. Somatic mutations were analyzed using the “Maftools” package.

In the TCGA-Advanced NSCLC cohort, the CIBERSORT algorithm [45], single-cell gene set enrichment analysis (ssGSEA) algorithm [46], Microenvironment Cell Populations-counter (MCPcounter) algorithm [47], and Estimation of Stromal and Immune cells in MAlignant Tumors using Expression data (ESTIMATE) algorithm [47] were used for quantify immune cells abundance.

### Enrichment analysis, drug susceptibility analysis and molecular docking simulation

Gene set enrichment analysis (GSEA) was performed according to the fold change of all genes in the TCGA-Advanced NSCLC cohort [48]. Differentially enriched KEGG pathways were identified using the Normalized Enrichment Score (NES) and adjusted p-value. The R package “clusterprofiler” was utilized for this analysis, with the “BH” method employed for p-value correction. Additionally, the cancer immunity cycle and 114 metabolic pathways were quantified using single-sample gene set enrichment analysis (ssGSEA) in the GSVA package.

Cancer Therapeutics Response Portal (CTRP) is a public database containing gene expression of cancer cell line and drug sensitivity information [49, 50]. The CTRP_2 data was downloaded as training sets using the R package “oncoPredict” [51]. The “calcPhenotype” package was employed to construct a ridge regression model that could be applied to the expression profile of the TCGA-Advanced NSCLC cohort and predict the half-maximal inhibitory concentration (IC50) of drugs in lung cancer patients.

Based on functional studies of seven prognostic genes, we screened five protein-coding genes in addition to *LINC00528* and *SLC16A4* for targeted drugs. Drug selection criteria focused on the cell death. Molecular docking simulations are used to predict the formation of stable complexes between large and small molecules. The structure of the proteins was obtained from the Protein Data Bank (https://www.rcsb.org/), and related small molecules were obtained from the zinc database (https://zinc.docking.org/). The Molecular Operating Environment (MOE) software was employed to recognize small molecule drugs and performed the molecular docking simulation to screen optimal docking posture for the anti-tumor drugs and target proteins according to the binding score.

### Cell culture and reverse transcription-quantitative polymerase chain reaction (RT-qPCR)

Two human NSCLC cell lines, A549 and NCI-H1299, and one human lung bronchial epithelial cell line (BEAS-2B) were obtained from the Stem Cell Bank, Chinese Academy of Sciences (Beijing, China). The NCI-H1299 cells were cultured in RPMI 1640 medium (Biosharp, Anhui, China); A549 cells were cultured in F12K medium (Biosharp, Anhui, China); and the BEAS-2B cells were cultured in DMEM medium (Biosharp, Anhui, China). All the culture solutions were supplemented with 10% fetal bovine serum (FBS, Gibco). Additionally, trypsin-EDTA (Biosharp, Anhui, China) was also needed to disperse the cells during the cell passage and collection. The cell cultures were maintained at a constant temperature of 37° C in a 5% CO_2_ atmosphere, providing optimal cell growth and viability conditions.

RT-qPCR experiments were conducted to validate the differential expression of seven PCD-related genes, including *CCL14, CPA3, CX3CR1, IKZF3, KIF21B, LINC00528,* and *SLC16A4* between NSCLC tumors cell lines (A549 and NCI-H1299) and normal lung bronchial epithelial cell line (BEAS-2B), and each. Total RNA was extracted using the TRIzol reagent (Invitrogen, USA) following the manufacturer’s protocol. Reverse RNA transcription to cDNA was obtained using PrimeScript® RT reagent Kit (TaKaRa, Shiga, Japan) in a 20 μl reaction mixture. The qPCR was performed with qPCR Kit (SYBR Premix Ex Taq) (TaKaRa, Shiga, Japan) in a 20 μl reaction mixture. Each gene expression reaction was performed in triplicates. The target gene expression was detected using the ABI7300 Fast instrument (Thermo Fisher Scientific, USA). The expression levels of each gene were normalized to the reference gene *GAPDH* and analyzed using the 2^(-ΔΔCT) method [52], which is a widely accepted approach in scientific research (ΔCT = CT (target gene) – CT (reference gene), ΔΔCT = ΔCT (NSCLC cells) − ΔCT (normal lung cell). The primer sequences are listed in Table 1, and the CT values of RT-qPCR are shown in Supplementary Table 9.

**Table 1:** Primer sequences utilized for RT-qPCR experiment in this study.

**Gene**	**Forward primer**	**Reverse primer**	**Size**
GAPDH	ATGGGGAAGGTGAAGGTCG	GGGGTCATTGATGGCAACAATA	152bp
SLC16A4	ACCACAAGTCTTACCTCATCCTCTG	TCAACCAGTACAGGCAGTATCAATG	145bp
LINC00528	GCTGAGCTTCTCTCCCTTTCCA	GTTTCCCAGAGCATAGGCAGTG	98bp
KIF21B	AAGAGCCGAGGATCAGAGAAGAC	ACATCATGGAGGACAGCAGGAG	128bp
IKZF3	ACCAAGCCATCAATAACGCCATC	GCTGCTGATAACTGGAACCATCTC	107bp
CX3CR1	CGTGGTCTTTGGGACTGTGTTC	GGCTTCTTGCTGTTGGTGAGG	108bp
CPA3	GAGTCCGAGAAAGAGACGAAAGC	TGGGAGTAGGAATGGAAGGTGATG	92bp
CCL14	TGCTGCTTCACCTACACTACCTAC	CTGGCTGTTGGTCTCATAGTAATCC	72bp

### Validation of the protein expression levels of the hub genes via the human protein atlas

To further verify the protein expression levels of *CCL14, CPA3, CX3CR1, IKZF3, KIF21B, LINC00528,* and *SLC16A4* in NSCLC and normal tissues, immunohistochemistry (IHC) data were downloaded from the Human Protein Atlas (HPA, http://www.proteinatlas.org).

### Statistical analysis

R software (4.2.1) and corresponding R packages were applied for all statistical analyses. For nonnormally distributed variables, significant quantitative differences between and among groups were determined by the Wilcoxon or Kruskal–Wallis tests, respectively. The Fisher test compared immune responses between other groups, and p < 0.05 was used as the significance threshold. Both univariate and multivariate Cox proportional hazards regression analyses were performed using the R package “survival”, and regression coefficients, hazards regression, 95% CI, and p-values were calculated. All KM survival curves were plotted using the R package “survminer”, and the two-sided log-rank test was used to assess differences between groups.

## Supplementary Material

Supplementary Figures

Supplementary Table 1

Supplementary Table 2

Supplementary Table 3

Supplementary Table 4

Supplementary Table 5

Supplementary Table 6

Supplementary Table 7

Supplementary Table 8

Supplementary Table 9
