# Supplementary material for: Revealing prognostic insights of programmed cell death (PCD)-associated genes in advanced non-small cell lung cancer
Source: Aging (Albany NY). 2024 May 8;16(9):8110–41. doi: 10.18632/aging.205807 (PMC11131998; doi:10.18632/aging.205807)
Supplement: Supplementary Figures [file aging-16-205807-s001.pdf]

SUPPLEMENTARY FIGURES

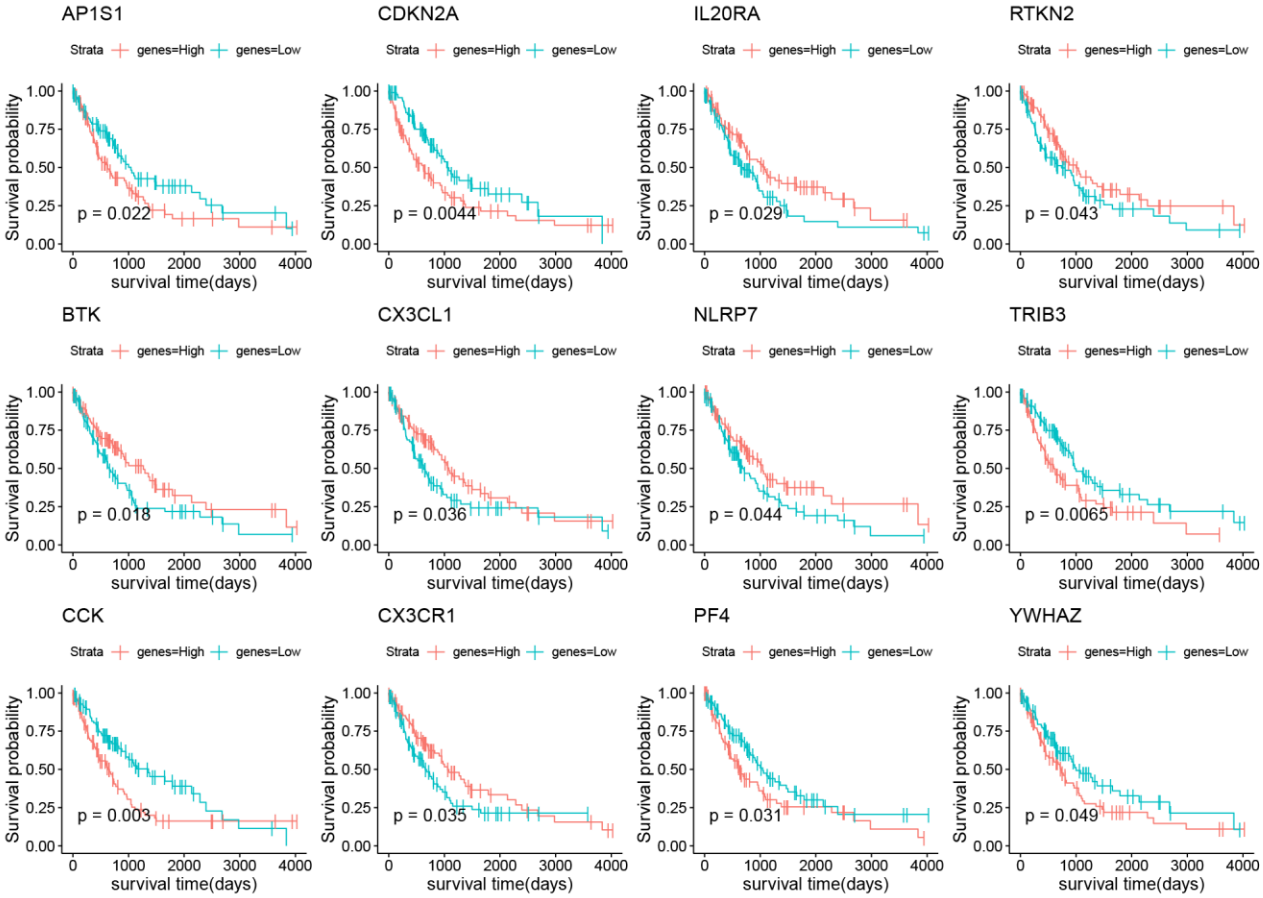

Supplementary Figure 1. The prognostic value of the 12 prognosis-related PCD genes.

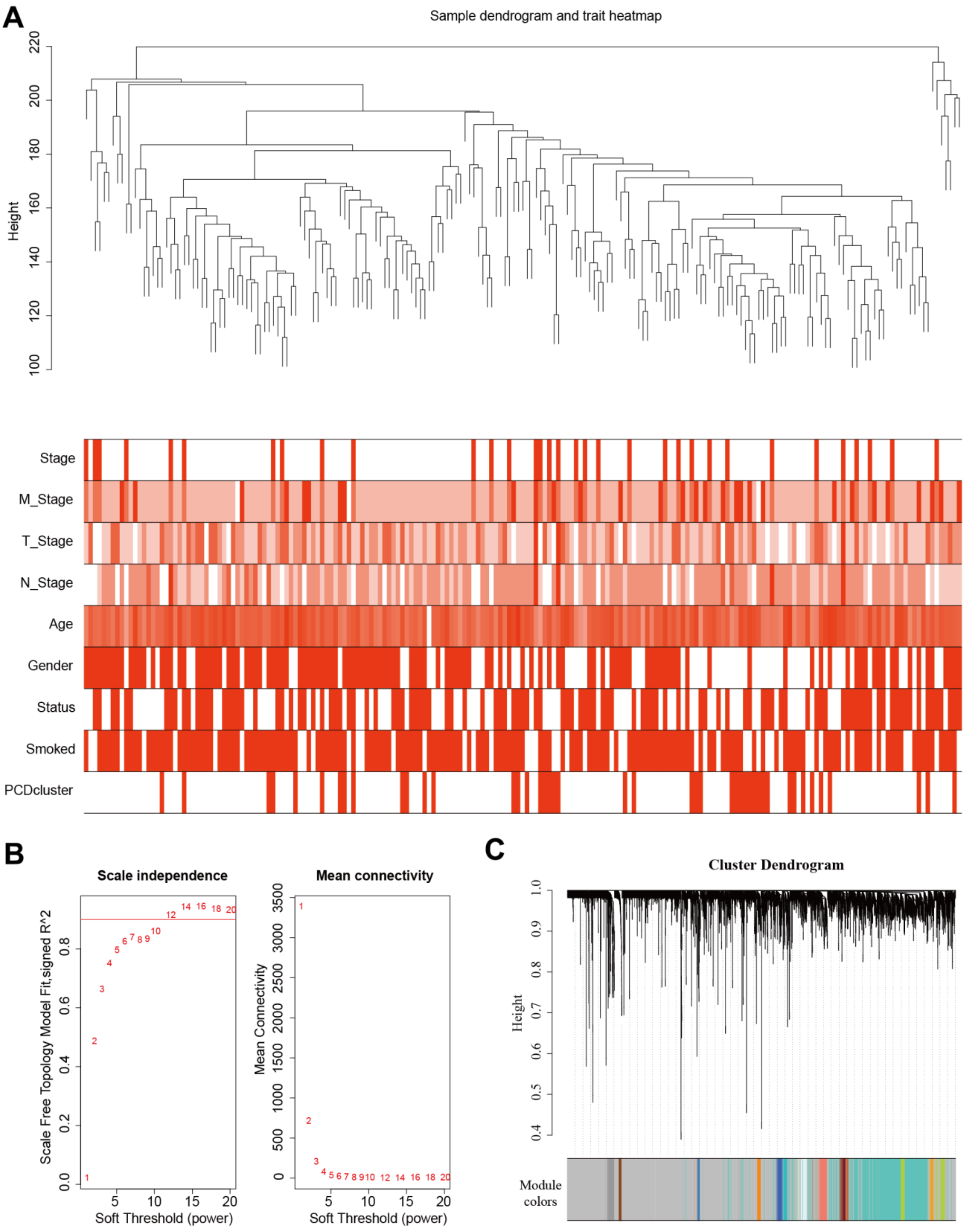

Supplementary Figure 2. Identification of modules by WGCNA. (**A**) Clustering dendrogram of TCGA-Advanced NSCLC samples and corresponding heatmap displaying clinical traits. The color band beneath the dendrogram represents the tissue trait status. (**B**) Evaluation of the scale-free fit index (left) and mean connectivity (right) across various soft threshold power values. The appropriate power value was determined for subsequent module identification. (**C**) Cluster dendrogram representing 39 distinct colored modules, based on a dissimilarity measure (1-TOM).

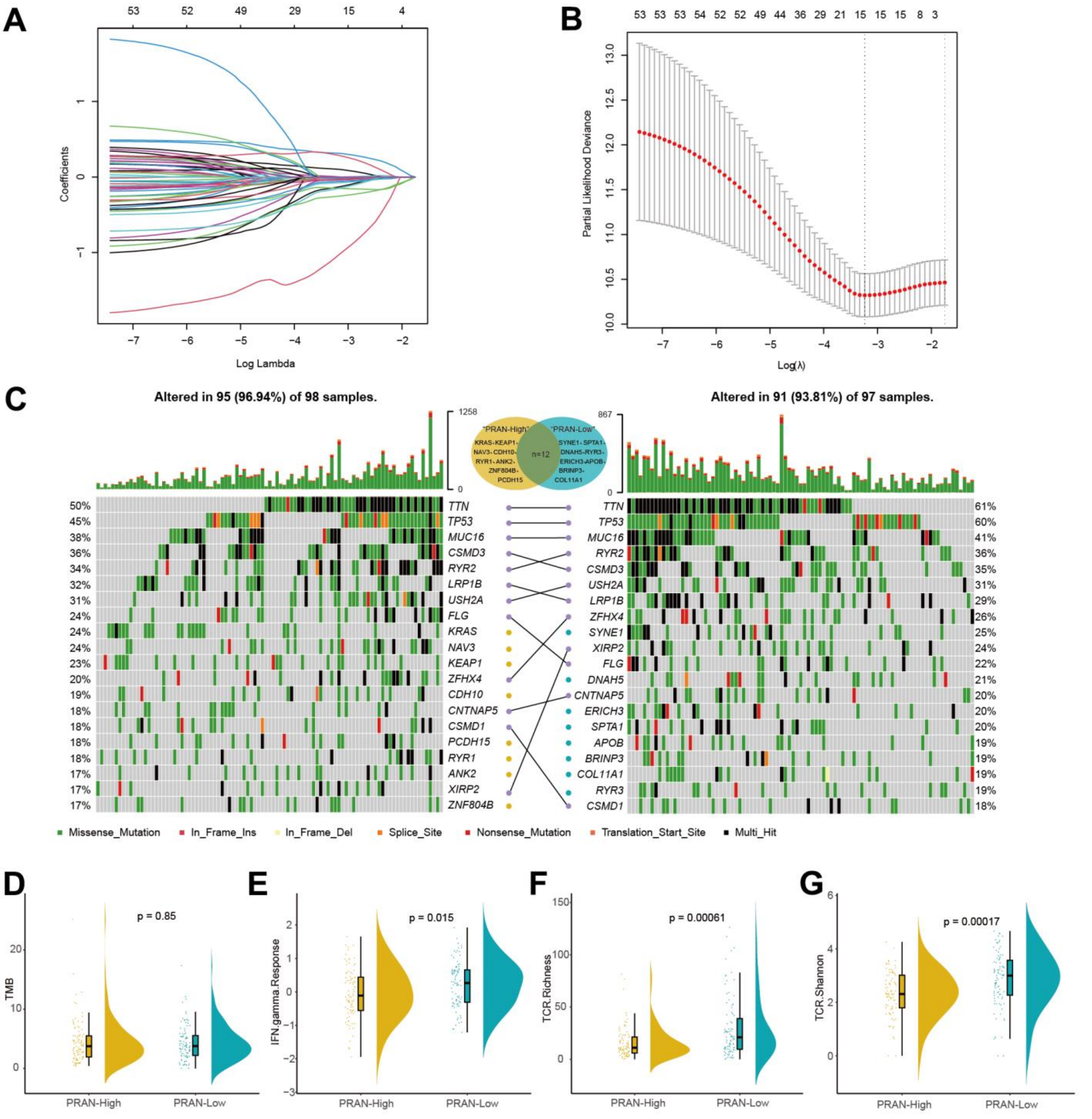

Supplementary Figure 3. The candidate genes screen by LASSO regression and association analysis of mutation profiles and immunological factors with PRAN risk score in the TCGA-Advanced NSCLC cohort. (**A**) The LASSO coefficient profiles of PCD-related genes. (**B**) Two dotted vertical lines are drawn at the optimal values according to the minimum criterion (right) and the Mean-Squared Error criterion (left). The horizontal axis represents the log value of lambda, and the vertical axis represents the Mean-Squared Error criterion of the independent variable. (**C**) The mutation profiles revealing the different mutation frequency between the two PRAN risk groups. (**D**) Box plot representing the comparison of Tumor Mutation Burden (TMB) levels between the two PRAN risk groups. (**E**) Box plot displaying the comparison of Interferon Gamma (IFN-γ) levels between the two PRAN risk groups. (**F**) Box plot showing the comparison of T-cell Receptor (TCR) richness levels between the two PRAN risk groups. (**G**) Box plot illustrating the comparison of T-cell Receptor (TCR) Shannon diversity index between the two PRAN risk groups.

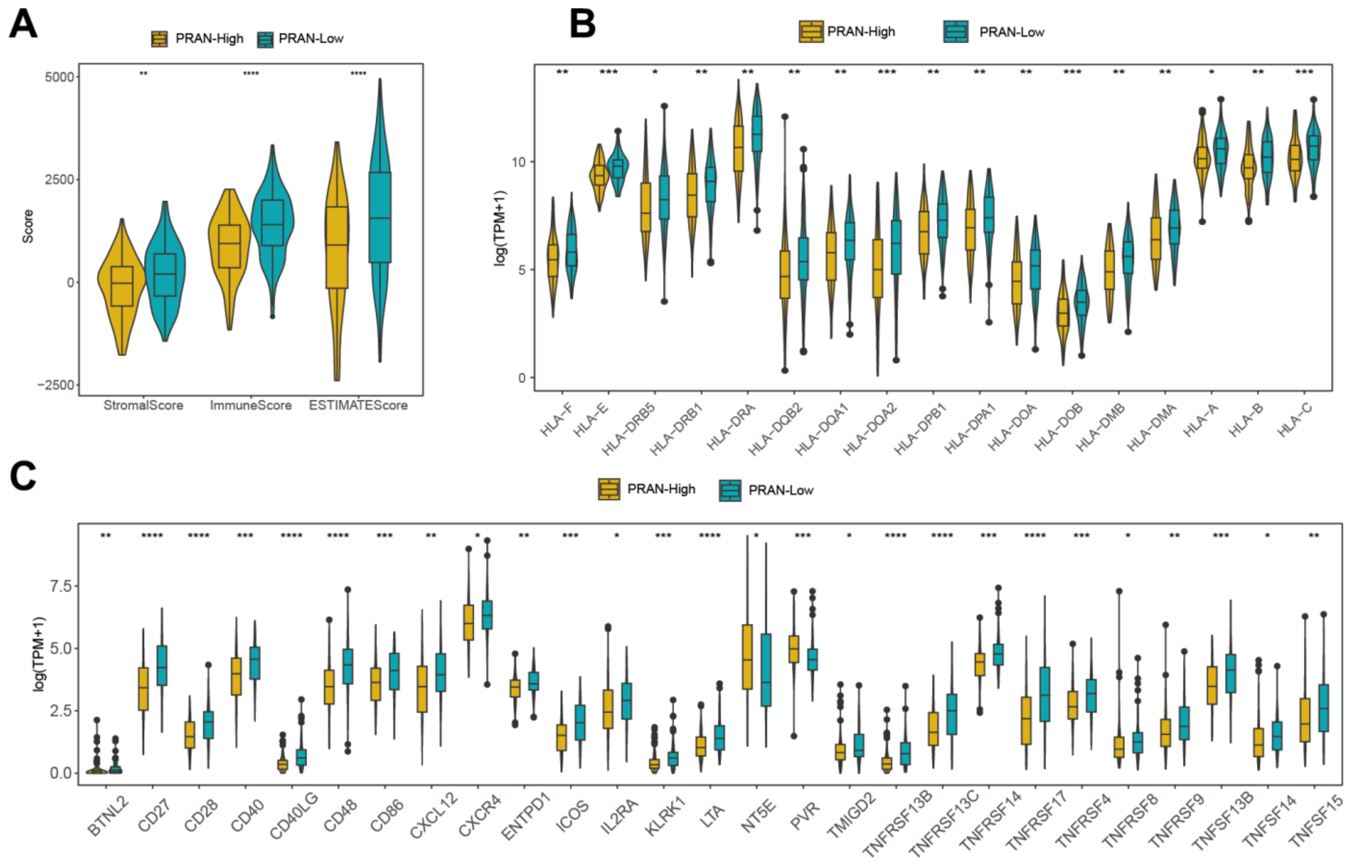

Supplementary Figure 4. Immune cell infiltration analysis between the two PRAN risk groups in the TCGA-Advanced NSCLC cohort. (**A**) Stromal score, immune score, and ESTIMATE score between PRAN-High and PRAN-Low groups. The expression of (**B**) HLA genes, and (**C**) immunostimulatory genes between PRAN-High and PRAN-Low groups. P value: * < 0.05; ** < 0.01; *** < 0.001; **** < 0.0001.

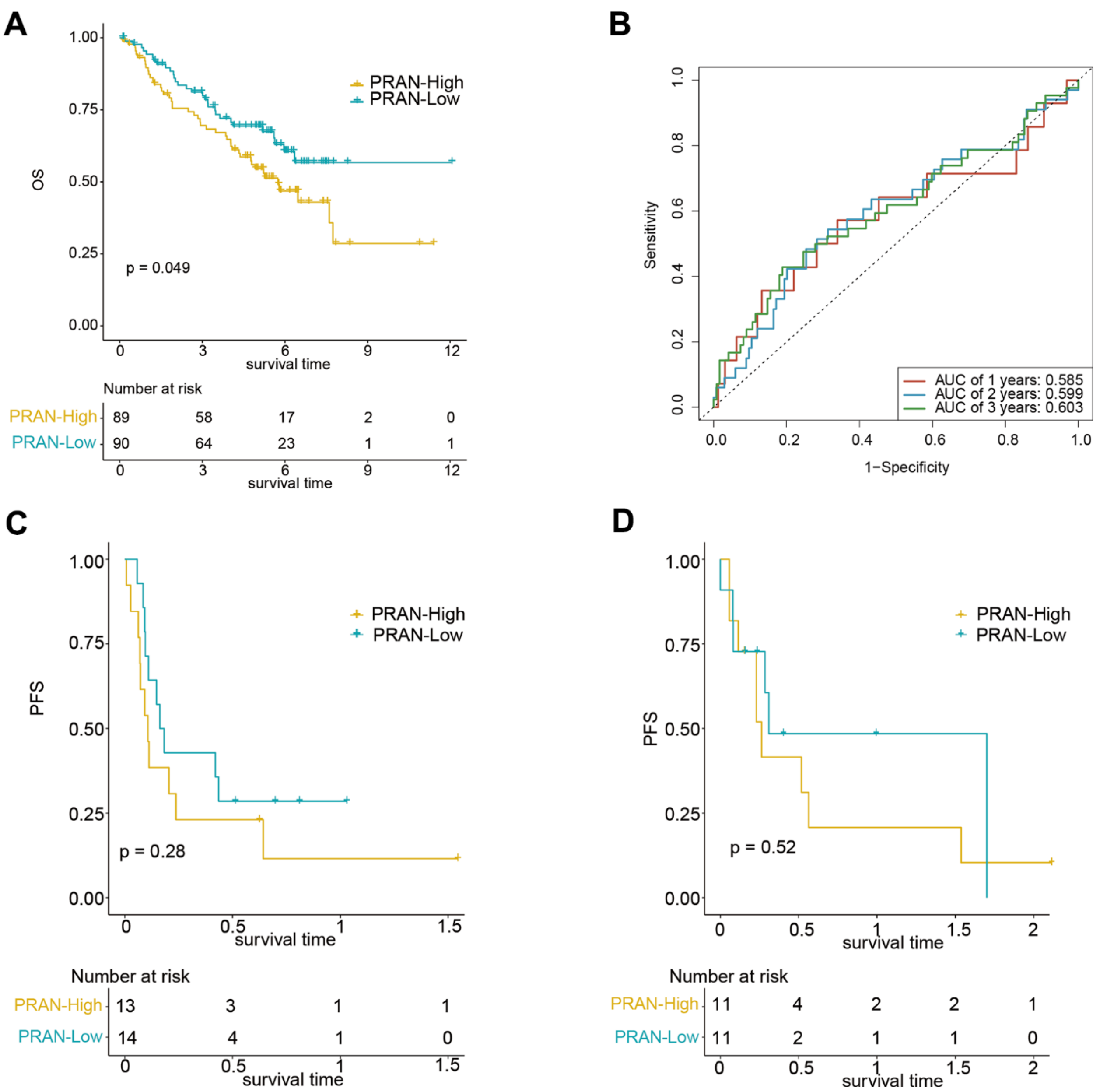

Supplementary Figure 5. The predicting performance validation of the PRAN risk model in multiple GEO cohorts. (**A**) Kaplan-Meier survival analysis in validation cohort GSE50081. (**B**) Time-dependent ROC curves between PRAN-High and PRAN-Low groups in validation cohort GSE50081. (**C**, **D**) Kaplan-Meier survival analysis in validation cohort GSE135222, and GSE93157.
