# Supplementary material for: Revealing prognostic insights of programmed cell death (PCD)-associated genes in advanced non-small cell lung cancer
Source: Aging (Albany NY). 2024 May 8;16(9):8110–41. doi: 10.18632/aging.205807 (PMC11131998; doi:10.18632/aging.205807)
Supplement: Supplementary Table 2 [file aging-16-205807-s003.pdf]

SUPPLEMENTARY TABLES

Supplementary Table 2. The sample information in TCGA-Advanced NSCLC cohort.

	TCGA_Advanced NSCLC					
	**Cluster1 (N=153)**	**Cluster2 (N=44)**	**P-value**	**High_risk (N=98)**	**Low_risk (N=99)**	**P-value**
**Age:**			0.213			1.000
<=65	65 (42.5%)	24 (54.5%)		44 (44.9%)	45 (45.5%)	
>65	88 (57.5%)	20 (45.5%)		54 (55.1%)	54 (54.5%)	
**Gender:**			0.010			0.267
Female	52 (34.0%)	25 (56.8%)		34 (34.7%)	43 (43.4%)	
Male	101 (66.0%)	19 (43.2%)		64 (65.3%)	56 (56.6%)	
**Stage:**			0.953			0.239
III	128 (83.7%)	36 (81.8%)		78 (79.6%)	86 (86.9%)	
IV	25 (16.3%)	8 (18.2%)		20 (20.4%)	13 (13.1%)	
**T_stage:**			0.665			0.769
T1	14 (9.15%)	6 (13.6%)		9 (9.18%)	11 (11.1%)	
T2	68 (44.4%)	19 (43.2%)		42 (42.9%)	45 (45.5%)	
T3	39 (25.5%)	8 (18.2%)		27 (27.6%)	20 (20.2%)	
T4	30 (19.6%)	10 (22.7%)		19 (19.4%)	21 (21.2%)	
TX	2 (1.31%)	1 (2.27%)		1 (1.02%)	2 (2.02%)	
**M_stage:**			0.254			0.052
Deficient	2 (1.31%)	1 (2.27%)		3 (3.06%)	0 (0.00%)	
M0	107 (69.9%)	25 (56.8%)		58 (59.2%)	74 (74.7%)	
M1	24 (15.7%)	8 (18.2%)		20 (20.4%)	12 (12.1%)	
MX	20 (13.1%)	10 (22.7%)		17 (17.3%)	13 (13.1%)	
**N_stage:**			0.896			0.418
N0	22 (14.4%)	8 (18.2%)		16 (16.3%)	14 (14.1%)	
N1	35 (22.9%)	11 (25.0%)		23 (23.5%)	23 (23.2%)	
N2	87 (56.9%)	22 (50.0%)		56 (57.1%)	53 (53.5%)	
N3	5 (3.27%)	2 (4.55%)		1 (1.02%)	6 (6.06%)	
NX	4 (2.61%)	1 (2.27%)		2 (2.04%)	3 (3.03%)	
**Tissue_origine:**			0.871			0.139
Lower lobe	51 (33.3%)	15 (34.1%)		26 (26.5%)	40 (40.4%)	
Middle lobe	9 (5.88%)	1 (2.27%)		4 (4.08%)	6 (6.06%)	
Other	17 (11.1%)	4 (9.09%)		11 (11.2%)	10 (10.1%)	
Upper lobe	76 (49.7%)	24 (54.5%)		57 (58.2%)	43 (43.4%)	
**Race:**			0.127			0.897
Asian	2 (1.31%)	3 (6.82%)		2 (2.04%)	3 (3.03%)	
Black or African						
American	14 (9.15%)	3 (6.82%)		9 (9.18%)	8 (8.08%)	
Unknown	39 (25.5%)	7 (15.9%)		21 (21.4%)	25 (25.3%)	
white	98 (64.1%)	31 (70.5%)		66 (67.3%)	63 (63.6%)	
**Smoking_Status:**			0.756			0.125
non-smoke Smoke	36 (23.5%)	12 (27.3%)		29 (29.6%)	19 (19.2%)	
	117 (76.5%)	32 (72.7%)		69 (70.4%)	80 (80.8%)	
**OS.time**		692		389		
**OS_status:**	468 [215;951]	[407;1177]	0.030	[144;728]	716 [420;1177]	<0.001
			0.139			0.003

Alive	62 (40.5%)	24 (54.5%)		32 (32.7%)	54 (54.5%)	
Dead	91 (59.5%)	20 (45.5%)		66 (67.3%)	45 (45.5%)	
**TMB:**			0.541			0.422
NA	1 (0.65%)	1 (2.27%)		0 (0.00%)	2 (2.02%)	
TMB<10	144 (94.1%)	41 (93.2%)		92 (93.9%)	93 (93.9%)	
TMB>=10	8 (5.23%)	2 (4.55%)		6 (6.12%)	4 (4.04%)	
