# Supplementary material for: Revealing prognostic insights of programmed cell death (PCD)-associated genes in advanced non-small cell lung cancer
Source: Aging (Albany NY). 2024 May 8;16(9):8110–41. doi: 10.18632/aging.205807 (PMC11131998; doi:10.18632/aging.205807)
Supplement: Supplementary Table 6 [file aging-16-205807-s007.pdf]

Supplementary Table 6. Interaction between target protein and candidate small molecules according to the molecular docking analysis.

**2Q8R:ZINC150338726(navitoclax)**								
Ligand		Receptor				Interaction	Distance	E(kcal/mol)
S	86	OE1	GLN	20	(H)	H-donor	3.87	-0.5
O	100	O	HOH	112	(G)	H-acceptor	2.93	-0.6
O	101	O	HOH	117	(H)	H-acceptor	2.91	-1.3
6-ring		CB	GLN	20	(H)	pi-H	4.26	-0.5
**4BD9:ZINC150338726(navitoclax)**								
Ligand		Receptor				Interaction	Distance	E(kcal/mol)
O	52	CA	GLU	136	(B)	H-acceptor	3.46	-0.6
5WN9:ZINC150338726(navitoclax)								
Ligand		Receptor				Interaction	Distance	E(kcal/mol)
6-ring		CA	LEU	184	(H)	pi-H	4.01	-0.5
**7BQV:ZINC000066099927(AZD1480)**								
Ligand		Receptor				Interaction	Distance	E(kcal/mol)
N	37	5-ring	TRP	380	(A)	H-pi	3.71	-0.7
6-ring		O	HOH	602	(B)	pi-H	3.3	-0.9
**7X4N:ZINC13476439(MG-132)**								
Ligand		Receptor				Interaction	Distance	E(kcal/mol)
N	38	OE2	GLU	411	(A)	H-donor	3.28	-1
O	18	NE	ARG	105	(A)	H-acceptor	3.16	-2
